# Up-regulation of the PI3K/AKT and RHO/RAC/PAK signalling pathways in CHK1 inhibitor resistant Eµ-Myc lymphoma cells

**DOI:** 10.1042/BCJ20220103

**Published:** 2022-10-14

**Authors:** Jill E. Hunter, Amy E. Campbell, Scott Kerridge, Callum Fraser, Nicola L. Hannaway, Saimir Luli, Iglika Ivanova, Philip J. Brownridge, Jonathan Coxhead, Leigh Taylor, Peter Leary, Megan S. R. Hasoon, Claire E. Eyers, Neil D. Perkins

**Affiliations:** 1 Newcastle University Biosciences Institute, Wolfson Childhood Cancer Research Centre, Newcastle University, Level 6, Herschel Building, Brewery Lane, Newcastle upon Tyne NE1 7RU, U.K.; 2 Centre for Proteome Research, Department of Biochemistry and Systems Biology, Institute of Systems, Molecular and Integrative Biology, University of Liverpool, Liverpool L69 7ZB, U.K.; 3 Newcastle University Clinical and Translational Research Institute, Preclinical In Vivo Imaging (PIVI), Faculty of Medical Sciences, Newcastle University, Newcastle Upon Tyne NE2 4HH, U.K.; 4 Bioinformatics Support Unit, Faculty of Medical Sciences, Newcastle University, Newcastle Upon Tyne NE2 4HH, U.K.

**Keywords:** CHK1 inhibitor, drug resistance, lymphoma, nuclear factor kappaB, p21-activated kinases, protein kinase B

## Abstract

The development of resistance and the activation of bypass pathway signalling represents a major problem for the clinical application of protein kinase inhibitors. While investigating the effect of either a c-Rel deletion or RelA^T505A^ phosphosite knockin on the Eµ-Myc mouse model of B-cell lymphoma, we discovered that both NF-κB subunit mutations resulted in CHK1 inhibitor resistance, arising from either loss or alteration of CHK1 activity, respectively. However, since Eµ-Myc lymphomas depend on CHK1 activity to cope with high levels of DNA replication stress and consequent genomic instability, it was not clear how these mutant NF-κB subunit lymphomas were able to survive. To understand these survival mechanisms and to identify potential compensatory bypass signalling pathways in these lymphomas, we applied a multi-omics strategy. With c-Rel^−/−^ Eµ-Myc lymphomas we observed high levels of Phosphatidyl-inositol 3-kinase (PI3K) and AKT pathway activation. Moreover, treatment with the PI3K inhibitor Pictilisib (GDC-0941) selectively inhibited the growth of reimplanted c-Rel^−/−^ and RelA^T505A^, but not wild type (WT) Eµ-Myc lymphomas. We also observed up-regulation of a RHO/RAC pathway gene expression signature in both Eµ-Myc NF-κB subunit mutation models. Further investigation demonstrated activation of the RHO/RAC effector p21-activated kinase (PAK) 2. Here, the PAK inhibitor, PF-3758309 successfully overcame resistance of RelA^T505A^ but not WT lymphomas. These findings demonstrate that up-regulation of multiple bypass pathways occurs in CHK1 inhibitor resistant Eµ-Myc lymphomas. Consequently, drugs targeting these pathways could potentially be used as either second line or combinatorial therapies to aid the successful clinical application of CHK1 inhibitors.

## Introduction

The development of targeted therapies has changed the face of cancer therapeutics and allowed for a more personalised approach to treatment based on the molecular basis of an individual's tumour. One of the kinases identified as a ‘druggable’ target is the checkpoint kinase, CHK1, and for this reason, CHK1 inhibitors (CHK1i) represent a potential new class of anti-cancer therapies, and are currently in clinical trials [1]. CHK1 plays a critical role in the response to DNA replication stress, which results from stalled DNA replication forks. In cancer cells, replication stress drives both genomic instability and clonal evolution [2–4]. Replication stress can be induced by a variety of mechanisms, including DNA damaging agents and by oncogenes such as MYC, driving hyper-DNA replication [2–4]. Critical regulators of the cellular response to DNA replication stress not only include CHK1, but also the kinase Ataxia Telangiectasia and Rad3 Related (ATR), which protect against tumourigenesis by promoting DNA repair [4,5]. However, once established, tumour cells can also become addicted to this pathway since it enables them to survive on-going, potentially lethal, genomic instability. Therefore, inhibiting key protein kinases, such as CHK1, provides a potential therapeutic strategy for specifically targeting tumours that have become dependent on their activity [6]. In a cancer cell adapted to cope with high levels of DNA replication stress, due to the activation of oncogenes such as MYC, for example, there appears to be an intimate association with tumour development and the increased rate of replication origin firing. This leads to the depletion of Replication protein A (RPA) and dNTPs, and therefore results in a large accumulation of stalled replication forks [1,7]. The cancer cell then has an increased reliance on CHK1 to prevent this ongoing DNA damage and fork collapse. Hence, inhibition of CHK1 is predicted to lead to the accumulation of unsustainable levels of damaged DNA that will result in mitotic catastrophe and ultimately tumour cell death in tumours with high levels of replication stress [8–11].

There are a number of known links between the NF-κB pathway and CHK1 signalling. These include the identification of the RelA Thr505 (T505) residue as a phosphosite that can be induced by DNA damaging agents such as cisplatin that activate CHK1, be modified by CHK1 *in vitro* and where its mutation to Ala confers resistance to inducers of DNA replication stress [12–14]. Phosphorylation of RelA T505 by CHK1 would provide a direct link between DNA replication stress signalling and NF-κB activity. However, it is not clear whether RelA T505 is a CHK1 target under all circumstances *in vivo* and other kinases may well phosphorylate this motif. The p50 NF-κB subunit has also been described as a direct target of CHK1, with its phosphorylation at Ser529 being reported to promote genomic stability [15]. The c-Rel NF-κB subunit has been shown to regulate the expression of Claspin, an adaptor protein required for activation of CHK1 by ATR gene expression [16]. We have used the Eµ-Myc model of B-cell lymphoma [17] as a system to study the role of NF-κB in the context of cancer with high levels of DNA replication stress. We have also seen, using mice with either a c-Rel gene knockout or with a RelA Thr 505 to Ala mutation (RelA^T505A^), that both Eµ-Myc/*cRel*^−/−^ and Eµ-Myc/RelA^T505A^ lymphomas exhibit lower levels of Claspin mRNA and protein expression [18].

We have previously shown that inhibition of CHK1, using the clinical candidate SRA-737 [19] and related inhibitor CCT244747 [18,20], is very effective at killing wild type (WT) Eµ-Myc lymphomas [18–20]. However, we also found that both Eµ-Myc/*cRel*^−/−^ and Eµ-Myc/RelA^T505A^ lymphomas, when reimplanted into wild type mice, had become resistant to CCT2447474 treatment [18,20] (Supplementary Figure S1A). Although, not yet reported for CHK1i, as their clinical use is thus far limited to trials, resistance to inhibitors targeting other kinases is a problem that is being frequently encountered in the clinic [21]. Our models of CHK1i resistance are classical examples of *de novo* resistance (reviewed in [21]). We therefore decided to investigate the mechanisms underpinning CCT244747 resistance in mutant NF-κB Eµ-Myc lymphomas to gain insights into how CHK1i resistance might arise *in vivo*.

Although there may be multiple different mechanisms that contribute to *de novo* resistance, one of the most common is intrinsic modulation of the drug target in the tumour. Indeed, we observed alterations in CHK1 activity and in both of our mutant NF-κB Eµ-Myc mice [18,20], with Eµ-Myc/*cRel*^−/−^ lymphomas exhibiting complete loss of CHK1 protein expression [20] (Supplementary Figure S1A). This resulted from an apparent combination of reduced CHK1 mRNA expression and down-regulation of the deubiquitinase USP1 that can function to promote CHK1 protein stability [20]. Other changes likely to affect ATR/CHK1 signalling were also seen in Eµ-Myc/*cRel*^−/−^ lymphomas, such as reduced Claspin expression, discussed above [18,20]. Since Claspin is associated with DNA replication forks and is required for ATR-dependent phosphorylation of CHK1 following DNA replication stress [22,23], a consequence of its loss would be reduced signalling through the ATR–CHK1 pathway. Confirming the intrinsic loss of CHK1 signalling in Eµ-Myc/*cRel*^−/−^ lymphomas, our proteomics analysis revealed that these cells have the characteristics of a WT Eµ-Myc lymphoma treated with an acute 8 h dose of CCT244747, with many down-regulated phosphopeptides from proteins linked to CHK1 being shared between these datasets [20].

In contrast, Eµ-Myc/*RelA^T505A^* lymphomas retain CHK1 protein expression but proteomics analysis revealed a reduced and altered response to CCT244747 treatment when compared with WT cells [18]. Here, the number of significantly changed phosphopeptides in Eµ-Myc/*RelA^T505A^* lymphomas after an acute dose of CCT244747 was ∼50% less than in wild type lymphomas (315 versus 625). Moreover, 77% of the CCT244747 induced phosphopeptide changes in WT Eµ-Myc were not seen in Eµ-Myc/*RelA^T505A^* lymphomas (481 out of 625), while conversely 54% (171 out of 315) of the changes in Eµ-Myc/*RelA^T505A^* lymphomas were not seen in WT Eµ-Myc cells [18]. We also observed loss of Claspin expression [18], together with reduced levels of USP1, albeit less dramatically than that seen in Eµ-Myc/*cRel*^−/−^ cells [20].

Since MYC-driven tumours become addicted to CHK1 signalling, an important question arising from our studies was how the mutant NF-κB subunit Eµ-Myc lymphomas that are deficient in this signalling pathway are still able to survive, especially as both models display earlier onset of disease [18,24] (Supplementary Figure S1B). One of the biggest contributing factors to kinase inhibitor resistance is activation of compensatory signalling pathways, so that the cell can bypass the need for the inhibited target, often referred to as ‘bypass signalling' [21]. There are consequently multiple strategies being successfully employed in the clinic that target these compensatory signalling pathways, either alone or in combination with the initial targeted agent. For example, in melanoma, a combination of BRAF and MEK inhibitors have shown efficacy [25]. Here, using an integrated multi-omics approach, we have characterised bypass pathways that become activated in our CHK1i resistant Eµ-Myc lymphoma models. We demonstrate that mutant NF-κB subunit Eµ-Myc lymphomas with CCT244747 resistance are now dependent upon bypass signalling *in vivo*. Targeting these bypass signalling pathways might therefore represent a potential therapeutic strategy to either treat patients developing CHK1i resistance in the clinic, or to develop combinatorial approaches to prevent this resistance arising in the first instance.

## Results

### Overlap between proteins and phosphopeptides up-regulated in Eµ-Myc/*cRel*^−/−^ lymphoma cells and WT Eµ-Myc lymphomas treated with CCT244747

We recently demonstrated that Eµ-Myc/*cRel*^−/−^ lymphoma cells reimplanted into wild type (WT) mice are highly resistant to treatment with the CHK1i, CCT244747, relative to WT Eµ-Myc controls [20]. This is mediated in part by a dramatic loss of CHK1 protein and signalling, thereby removing the target of the inhibitor [20] (Supplementary Figure S1A). However, this raised the question of how these cells were surviving and coping with ongoing DNA replication stress (Supplementary Figure S1B). Since activation of compensatory signalling pathways is a major contributing factor in the development of resistance to kinase inhibitors [26–31], and often goes hand-in-hand with the tumour cell having bypassed the need for the drug target, we therefore investigated whether this was occurring in Eµ-Myc/*cRel*^−/−^ lymphoma cells.

To gain insight into the response to CCT244747, the development of *de novo* CCT244747 resistance and the intrinsic differences between our Eµ-Myc B-cell lymphoma models we performed a comprehensive (phospho) proteomic analysis using tandem mass tag (TMT)-based isobaric labelling to quantify relative changes in both total protein levels and phosphopeptide abundance [18,20]. This analysis used either WT, cRel^−/−^ or RelA^T505A^ Eµ-Myc lymphomas, reimplanted into wild type (WT) mice. After a period to allow lymphomas to develop, mice were then treated with single dose of CCT244747 or a vehicle control for 8 h. As reported [18,20], of the ∼4000 proteins identified overall at a 1% false discovery rate (FDR), ∼2500 were quantified in at least three biological replicates (Supplementary Data File S1). At the phosphopeptide level, we identified a total of over 6500 phosphopeptides, quantifying ∼3350 in at least three replicates (Supplementary Data File S1).

**Figure 1.**
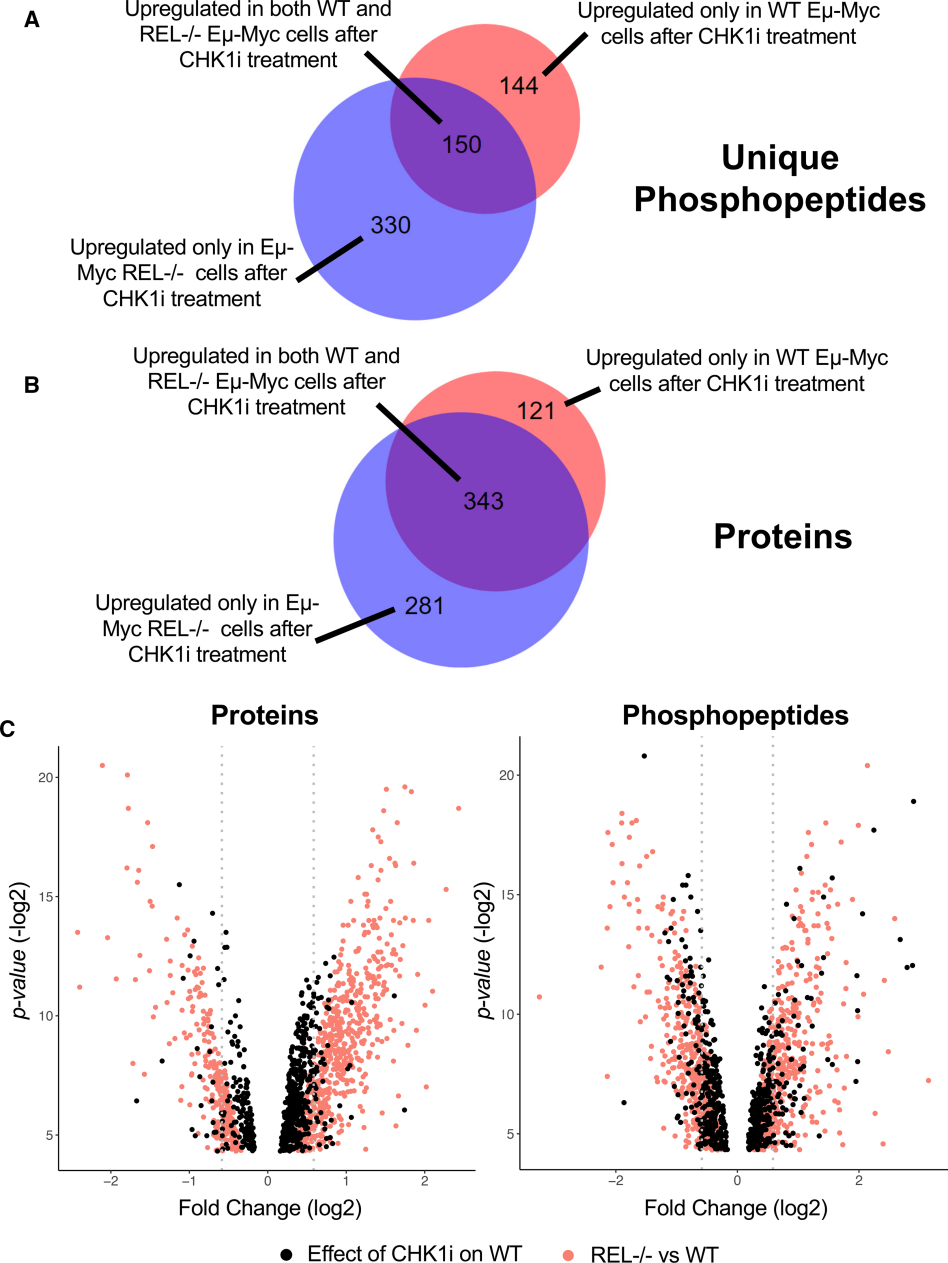
Analysis of up-regulated phosphopeptides in Eµ-Myc/*c-Rel*^−/−^ lymphomas and Eµ-Myc WT lymphomas following acute CCT244747 treatment. (**A**) Venn diagram illustrating that of the 294 unique up-regulated phosphopeptides seen in Eµ-Myc WT tumours following acute CCT244747 treatment, 150 were also up-regulated in Eµ-Myc/c-Rel^−/−^ lymphoma cells without inhibitor treatment. (**B**) Venn diagram illustrating that of the 464 up-regulated proteins seen in Eµ-Myc WT tumours following acute CCT244747 treatment, 343 were also up-regulated in Eµ-Myc/c-Rel^−/−^ lymphoma cells without inhibitor treatment. (**C**) Overlaid volcano plots showing fold changes (log_2_) of proteins and phosphopeptides in both Eµ-Myc WT tumours following acute CCT244747 treatment (black dots) and in Eµ-Myc/c-Rel^−/−^ lymphoma cells (red dots) compared with WT lymphomas (both without CCT244747 treatment). Only changes with a *P*-value of <0.05 are shown.

To identify up-regulated components potentially representative of bypass signalling pathways that compensate for loss of CHK1 in Eµ-Myc/*cRel*^−/−^ lymphoma cells [20], we further interrogated our (phospho)proteomic datasets (Supplementary Data File S1), focusing on those proteins and phosphopeptides that were elevated in Eµ-Myc/*cRel*^−/−^ relative to WT Eµ-Myc lymphomas. These analyses revealed 517 up-regulated phosphopeptides (*P* < 0.05), representing 480 unique phosphopeptides correlating to 297 confidently identified unique phosphosites (with a ptmRS value, a confidence localisation score for post-translational modifications (PTMs), of ≥0.998), together with 624 up-regulated proteins (*P* < 0.05) in Eµ-Myc/*cRel*^−/−^ lymphomas with no CCT244747 treatment, compared with wild type controls (Supplementary Data Files S1, S2). We had previously observed that there was considerable overlap in those phosphopeptides and proteins down-regulated between the wild type Eµ-Myc lymphomas after CCT244747 treatment and the Eµ-Myc/*cRel*^−/−^ lymphomas without any treatment [20]. Therefore, we were interested in whether the up-regulated proteins and phosphopeptides in Eµ-Myc/*cRel*^−/−^ lymphomas similarly overlapped with the changes observed upon treatment of WT Eµ-Myc lymphomas with CCT244747. This analysis would indicate the extent to which these changes were potentially being driven by loss of CHK1. Similar to this previous analysis there was considerable overlap between these datasets. Of the 294 unique phosphopeptides and 464 proteins elevated in wild type Eµ-Myc lymphomas in response to CCT244747, 51% and 74%, respectively were also up-regulated in Eµ-Myc/*cRel*^−/−^ cells without treatment (Figure 1A,B, Supplementary Data File S3). Manual inspection of the data revealed that many apparent unique phosphopeptides seen to increase in Eµ-Myc/*cRel*^−/−^ lymphomas (*P* value < 0.05) were also increased in the WT Eµ-Myc lymphomas but that the data fell below the threshold for significance at a *P* value >0.05 and so were not included in this analysis.

The magnitude of the increases in both phosphopeptides and proteins seen in the CCT244747 treated wild type cells appeared generally lower than the differences between Eµ-Myc/*cRel*^−/−^ and WT lymphomas (Figure 1C). Moreover, correlation analysis of the individual phosphopeptides and proteins confirmed that those up-regulated in one dataset were generally also up-regulated in the other, although some outliers with opposing effects were seen (Supplementary Figure S2). Nonetheless, this further confirmed the similarities between Eµ-Myc/*cRel*^−/−^ lymphomas and their wild type equivalents following CHK1 inhibition. Overall, these data suggested that loss of CHK1 activity results in activation of potential compensatory bypass pathways.

### AKT signalling is up-regulated in Eµ-Myc/*cRel*^−/−^ lymphoma cells

To determine whether analysis of up-regulated phosphosites in Eµ-Myc/*cRel*^−/−^ lymphomas could reveal information about the activity of compensatory cell signalling pathways we first performed a manual interrogation of the data. The most highly induced (8.8 fold, *P*-value = 6.68 × 10^−03^) putative phosphosite in Eµ-Myc/*cRel*^−/−^ lymphomas relative to WT Eµ-Myc controls was S116 in PEA-15, (Supplementary Figure S3A, Supplementary Data Files S1, S2). In total, 3 phosphopeptides where the S116 motif was identified as the putative phosphosite were detected in our proteomics analysis, deriving from two trypsin missed cleavage sites (YKDIIRQP**S**EEEIIK, where missed cleavages are underlined). Although all were elevated in the Eµ-Myc/*cRel*^−/−^ lymphomas, the comparatively smaller increase (1.7 *c.f.* 8.8 fold change) in the relative abundance of the fully tryptic phosphopeptide suggests additional localised structural differences that change trypsin accessibility and thus the ability to generate the ‘limit’ peptide. No other PEA-15 phosphopeptides were identified.

PEA-15 is an anti-apoptotic protein and regulator of MAP kinase (MAPK) signalling [32]. PEA-15 can bind to and inhibit the nuclear translocation of the MAPKs ERK1 and ERK2, with phosphorylation at S104 and S116 disrupting this interaction hence promoting ERK1/2 activity [33]. PEA-15 S116 has also been described as a target of AKT1 (Protein Kinase B), a consequence of which is to stabilise the anti-apoptotic activity of PEA-15 [34]. We therefore performed further manual analysis of the phosphopeptide data, cross referencing up-regulated putative phosphosites in the Eµ-Myc/*cRel*^−/−^ lymphomas with a list of known AKT target sites available on the Cell Signalling Technology website (https://www.cellsignal.co.uk/learn-and-support/reference-tables/pi3k-akt-substrates-table). From this we also identified AKT1S1 (PRAS40) T247 (T246 in human, 2.3 fold, *P*-value = 3.51 × 10^−04^) and HSPB1 (HSP27) S86 (S82 in human, 1.8 fold, *P*-value = 1.07 × 10^−02^) as additional known AKT1 targets [35,36] (Supplementary Figure S3A, Supplementary Data File S2). In our total phosphoproteomic dataset we detected three other AKT1S1 and two other HSPB1 phosphopeptides but these did not show any significant differences between different Eµ-Myc samples, suggesting the up-regulation of phosphorylation of the putative AKT sites was specific and a consequence of altered kinase activity (Supplementary Data File S2). In addition, we identified 12 other proteins that have been described as AKT targets but where the listed site of phosphorylation differed from the ones that we identified in our study (Supplementary Figure S3A, Supplementary Data File S2). We also observed increased phosphorylation of a number of kinases in the Eµ-Myc/*cRel*^−/−^ lymphomas (Supplementary Figure S3B, Supplementary Data File S2)). These included p38 MAPK (MAPK14) at Y182 (2.1 fold, *P*-value 1.47 × 10^−02^) that together with T180 (not detected) is required for its activation [37]. We also detected putative phosphorylation of MAP4K1 at S370 (1.4 fold, *P*-value 4.20 × 10^−02^) and MAP4K4 at S701 (1.6 fold, *P*-value 1.73 × 10^−03^), both of which function upstream of the JNK signalling pathway [38,39]. Taken together, this manual interrogation revealed that Eµ-Myc/*cRel*^−/−^ lymphoma cells have undergone a complex rewiring of their cell signalling pathways, potentially involving up-regulation of AKT, JNK, ERK and p38 MAPK activity.

**Figure 2.**
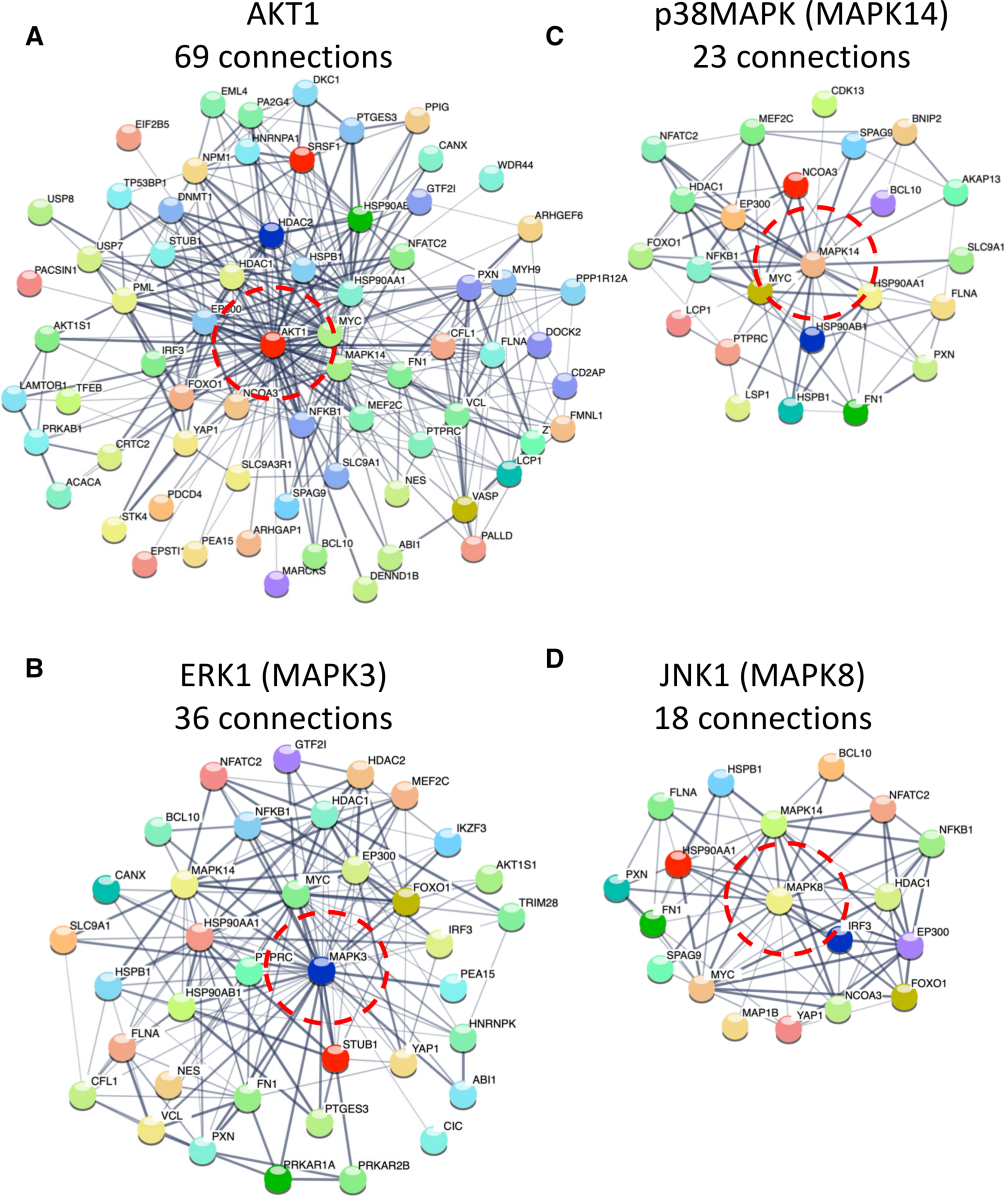
STRING analysis showing proteins with up-regulated phosphorylation in Eµ-Myc/*c-Rel*^−/−^ that are linked to AKT1, ERK1, JNK1 and p38MAPK. String analysis was performed on all proteins with up-regulated phosphopeptides in Eµ-Myc/c-Rel^−/−^ lymphomas relative to WT Eµ-Myc lymphomas. AKT1, ERK1 (MAPK3) and JNK1 (MAPK8) were manually added to the list to identify potential functional links. p38MAPK (MAPK14) was already in the list of proteins. Further string analysis was then performed using just these lists of proteins (Supplementary Fig. S4) to generate images showing just the networks linked to (**A**) AKT1, (**B**) ERKa (MAPK3), (**C**) p38 MAPK (MAPK14) and (**D**) JNK1 (MAPK8). Analysis shown was performed under STRING medium confidence setting. Numbers of connections are shown and AKT1, ERK1, JNK1 and p38 MAPK are circled in red. See also Supplementary Figures S3, S5 and Supplementary Data File S4.

Although we cannot rule out that the other kinases we see being phosphorylated play an important role in the phenotype of Eµ-Myc/*cRel*^−/−^ lymphomas, we decided to focus on potential changes to the AKT, JNK, ERK and p38 MAPK signalling pathways as these constitute highly studied and druggable targets. First, we looked more broadly at links between these kinases and the proteins with up-regulated phosphopeptides in Eµ-Myc/*cRel*^−/−^ lymphomas by performing STRING analysis (https://string-db.org/) [40]. STRING analysis uses a variety of databases and sources of information to either identify or predict functional interactions between the members of an inputted list of proteins. We performed this analysis using the list of proteins with an Eµ-Myc/*cRel*^−/−^ lymphoma up-regulated phosphopeptide (relative to WT Eµ-Myc cells) and manually added in either AKT1, ERK1 or JNK1 to identify any potential links. p38 MAPK (MAPK14) was already in the list and so did not need to be manually included. Although STRING analysis is not capable of predicting whether any proteins linked to these kinases is actually a substrate for phosphorylation, it can identify the existence of a potentially wider regulatory network. This network analysis revealed that AKT1 had the most potential links (69 medium, 29 high, from 514 proteins input), followed by ERK1 (36/19), p38 MAPK (23/11) and JNK1 (18/11) (Figure 2, Supplementary Figures S1, S3, Supplementary Data File S4).

**Figure 3.**
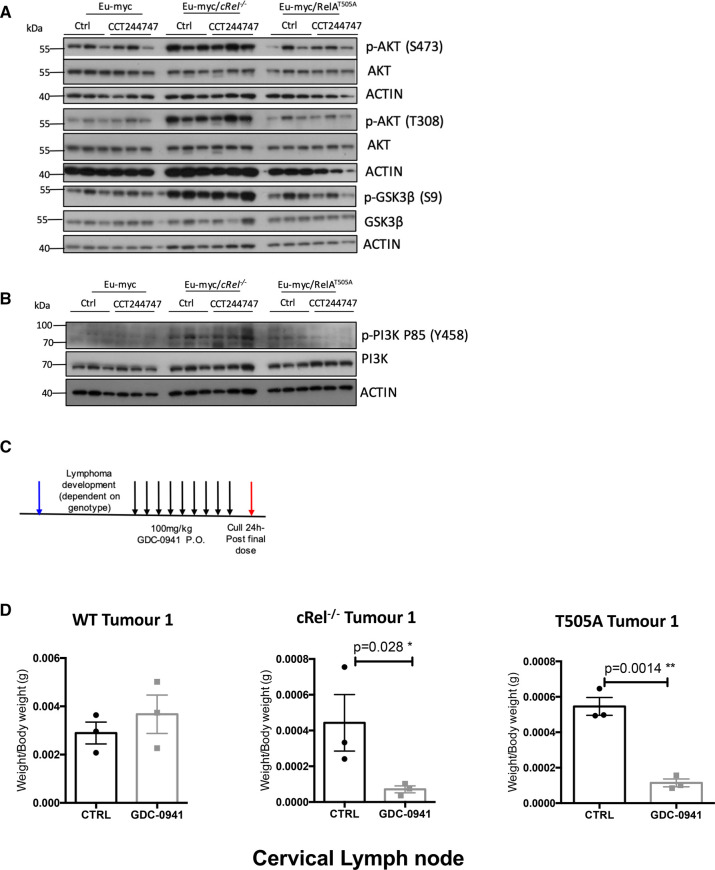
Up-regulation of PI3K/AKT activity in Eµ-Myc *c-Rel*^−/−^ lymphomas. (**A**) Western blot analysis of phospho-Ser473 AKT, phospho-Thr308 AKT, AKT, phospho-Ser9 GSK3B, GSK3B or actin in snap frozen tumour extracts prepared from re-implanted Eµ-Myc Eµ-Myc *c-Rel*^−/−^ and Eµ-Myc/RelA^T505A^ tumours from inguinal lymph nodes 8 h following a single dose of CCT244747. The data shows that the AKT pathway is highly active in Eµ-Myc/*c-Rel*^−/−^ tumours, and to a lesser extent in the Eµ-Myc/RelA^T505A^ cells. (**B**) Western blot analysis of phospho-Tyr458 PI3K, PI3K, or actin in snap frozen tumour extracts prepared from re-implanted Eµ-Myc and Eµ-Myc/*c-Rel*^−/−^ tumours mouse inguinal lymph nodes 8 h following a single dose of CCT244747. The data shows that the PI3K/AKT pathway is highly in Eµ-Myc*/c-Rel*^−/−^ tumours. (**C**) Schematic diagram illustrating the PI3Ki *in vivo* study in Eµ-Myc, Eµ-Myc/*c-Rel*^−/−^ and Eµ-Myc/RelA^T505A^ mice. 6 week old C57Bl/6 WT mice were implanted with either Eµ-Myc, Eµ-Myc/*c-Rel*^−/−^ or Eµ-Myc/RelA^T505A^ (blue arrow) and once tumours became palpable were treated with either 100 mg/kg GDC-0941/Pictilisib p.o or vehicle control once daily for 9 days (black arrows). Mice were euthanised 24 h after the final dose (red arrow) and tumour burden assessed. (**D**) Scatter plot showing the response of reimplanted Eµ-Myc Eµ-Myc/*c-Rel*^−/−^ and Eµ-Myc/RelA^T505A^ tumours to GDC-0941/Pictilisib in the cervical lymph node tumour site. Each tumour was implanted into six syngeneic recipient C57Bl/6 mice, three were treated with GDC-0941/Pictilisib (100 mg/kg p.o), and three with vehicle control, for 9 days once tumours became palpable. A response was defined as a significant reduction (or increase) in tumour burden (*P* < 0.05) using unpaired Student's *t*-tests. WT Eµ-Myc showed little response to GDC-0941/Pictilisib whereas the Eµ-Myc/*c-Rel*^−/−^ and Eµ-Myc/RelA^T505A^ tumours were reduced by GDC-0941/Pictilisib.

To confirm that AKT activity was indeed up-regulated in Eµ-Myc/*cRel*^−/−^ lymphomas, as suggested by the elevated levels of putative substrate phosphosites and the network analysis, we examined AKT phosphorylation on Ser473 and Thr308 by western blot. These antibodies recognise AKT1, 2 and 3 with phosphorylation of these sites being a marker for ‘full’ AKT activity [41]. Although we observed phosphorylation of S473 and T308 on AKT in WT cells, immunoreactivity increased significantly in the Eµ-Myc/*cRel*^−/−^ lymphomas (Figure 3A, Supplementary Figure S6A) suggesting elevated activity levels. AKT activation is known to induce a pro-survival response by phosphorylating GSK3β on Ser9, which inhibits its activity, a site which we also observe to be enhanced in the c-Rel^−/−^ cells (Figure 3A). The AKT1-activating Phosphoinositide 3-kinase (PI3K) pathway was also up-regulated in Eµ-Myc/*cRel*^−/−^ lymphomas, with elevated levels of phosphorylation of the p85 (Y458) subunit being observed (Figure 3B). Taken together, these data demonstrate that the PI3K/AKT pathway is up-regulated in Eµ-Myc/*cRel*^−/−^ lymphomas. In contrast, these markers of PI3K/AKT pathway activity were not elevated in Eµ-Myc/*RelA^T505A^* lymphomas.

We also observed an increase in T202/Y204 ERK1/2 phosphorylation in Eµ-Myc/*cRel*^−/−^ lymphomas (Supplementary Figure S6B,C). Phosphorylation of these sites is required for ERK1/2 activation [42] and this is consistent with up-regulation of signalling through this pathway predicted from the phosphopeptide analysis above. There was also an increase in PEA15 levels, discussed above as a potential AKT substrate (Supplementary Figure S3A) and regulator of ERK/MAPK signalling (Supplementary Figure S6D) [43]. In contrast, there was reduced activation site phosphorylation for both JNK1/2 and p38 MAPK in Eµ-Myc/*cRel*^−/−^ lymphomas (Supplementary Figure S6B,C). We had seen an increase in p38 MAPK phosphorylation at Y182 in the phosphopeptide data (Supplementary Figure S3B) but did not detect phosphorylation at T180 (Supplementary Data File S1). Therefore, the decrease in signal seen here possibly results from the antibody used specifically recognising simultaneous T180 and Y182 phosphorylation. Also included in our western analysis were extracts from Eµ-Myc/*RelA^T505A^*lymphomas. Although a basal level of phosphorylation of the AKT1/2, ERK1/2, JNK1/2 or p38 MAPK activation sites was seen, indicating that these pathways are active in these cells, no significant and consistent difference were seen with WT Eµ-Myc lymphomas (Figure 3A,B, Supplementary Figure S6B,C)

Taken together with our previous analysis of down-regulated (phospho)proteins [20], these data demonstrate that Eµ-Myc/*cRel*^−/−^ lymphomas have undergone significant rewiring of their signalling pathways, specifically hyper-activation of the PI3K/AKT and ERK pathways, which are known to inhibit apoptosis and cell cycle arrest [44,45], potentially explaining how these tumours are surviving loss of CHK1.

### Inhibition of PI3K/AKT signalling provides an alternative therapeutic strategy in lymphomas exhibiting resistance to CHK1 inhibitors

We hypothesised that if up-regulation of the PI3K/AKT pathway allowed Eµ-Myc/*cRel*^−/−^ lymphomas to survive in the absence of CHK1, then targeting this pathway could be a good therapeutic strategy for tumours that have developed CHK1i resistance. We therefore evaluated the effectiveness of the PI3K inhibitor, GDC-0941/Pictilisib [46] *in vivo,* determining its effect on the growth of three transplanted WT Eµ-Myc, Eµ-Myc/*cRel*^−/−^ and Eµ-Myc/*RelA^T505A^* tumours. As previously performed with CCT244747 [18,20], each tumour was implanted into six syngeneic C57Bl/6 recipient mice and three were treated orally with GDC-0941 once a day for nine days, while three received a vehicle control (Figure 3C). After treatment, we observed a striking reduction in lymphoid tumour burden in all mice re-implanted with Eµ-Myc/*cRel*^−/−^ lymphoma cells and treated with GDC-0941 (Figure 3D, Supplementary Figure S7A,B). In contrast, in all of the WT Eµ-Myc tumours, no significant reduction in lymphoid tumour burden was seen after GDC-0941 treatment. Surprisingly, even though Eµ-Myc/*RelA^T505A^* lymphomas did not display significant up-regulation of PI3K or AKT activity by western blot, they were still sensitive to GDC-0941 treatment (Figure 3D, Supplementary Figure S7), suggesting that the down-regulation of CHK1 activity we see in these cells [18] creates a dependence on the existing PI3K/AKT activity they possess (Figure 3A, Supplementary Figure S6A). These data confirmed that PI3K/AKT signalling critically compensates for loss of CHK1 activity in both Eµ-Myc/*cRel*^−/−^ and Eµ-Myc/*RelA^T505A^* lymphomas. Targeting this pathway may well, therefore, represent a viable therapeutic strategy for the treatment of CHK1i resistant tumours in human patients.

### Rac/Rho GTPase signalling is up-regulated in Eµ-Myc/RelA^T505A^ lymphoma cells

Although the data above indicated that Eµ-Myc/RelA^T505A^ lymphoma cells had become dependent upon their existing levels of PI3K/AKT signalling, this pathway was not up-regulated relative to WT Eµ-Myc lymphoma cells (Figure 3A,B, Supplementary Figure S6A). We were therefore interested in whether other compensatory bypass pathways might have become constitutively up-regulated in the Eµ-Myc/RelA^T505A^ lymphoma cells. Analysis of the phosphoproteomics data revealed that there was very little overlap between the up-regulated phosphopeptides (*P* value <0.05) seen in Eµ-Myc/RelA^T505A^ and Rel^−/−^ lymphoma cells relative to WT Eµ-Myc controls, with only 25 out of 234 or 480 being identical, respectively (Supplementary Figure S8A). Furthermore, neither GOTERM analysis or functional annotation clustering proved informative from the perspective of identifying potential druggable targets, with proteins associated with mRNA processing and splicing being highly enriched (not shown).

**Figure 4.**
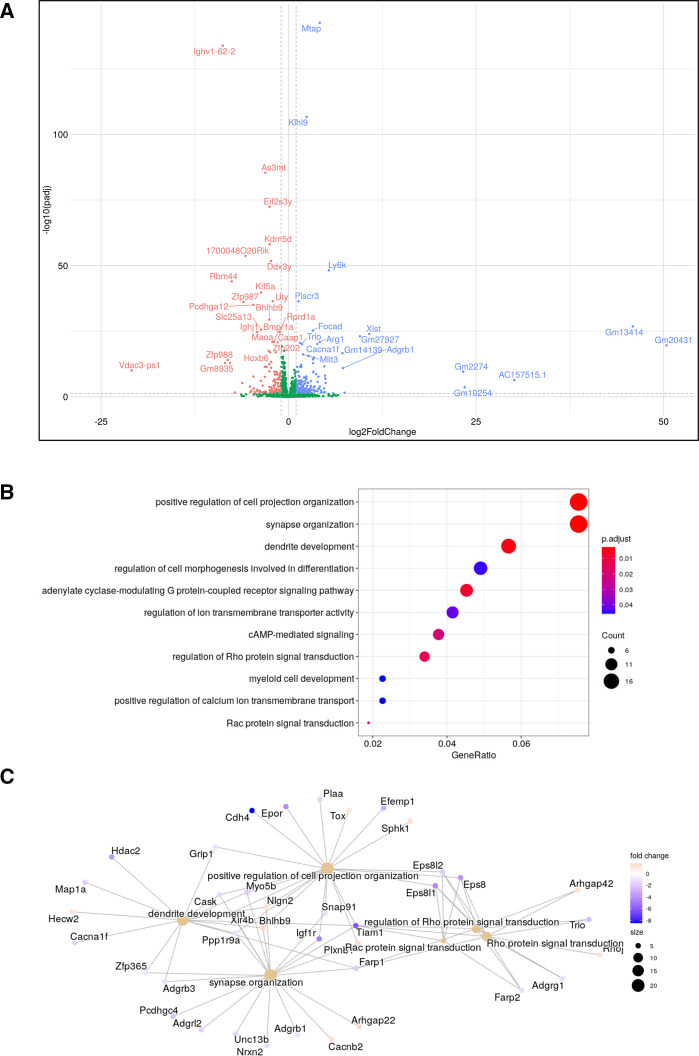
Up-regulation of a RHO/RAC pathway gene expression signature in Eµ-Myc/RelA^T505A^ lymphoma cells. (**A**) Volcano plot comparing changes in mRNA transcripts between Eµ-Myc WT and Eµ-Myc/RelA^T505A^ lymphoma cells with no CCT244747 treatment. (**B**) Dotplot showing the top enriched GO terms (gene ratio on *x*-axis, dot sizes = gene number, coloured by adj pval) in the Eµ-Myc/RelA^T505A^ RNA-Seq analysis. GO enrichment analysis was performed using the enrichGO function in the package clusterProfiler with an FDR cutoff of 0.05. (**C**) RHO/RAC pathway components up-regulated Eµ-Myc/RelA^T505A^ RNA-Seq analysis displayed in a cnetplot, which was created from the enrichGO GO term analysis in (**B**).

**Figure 5.**
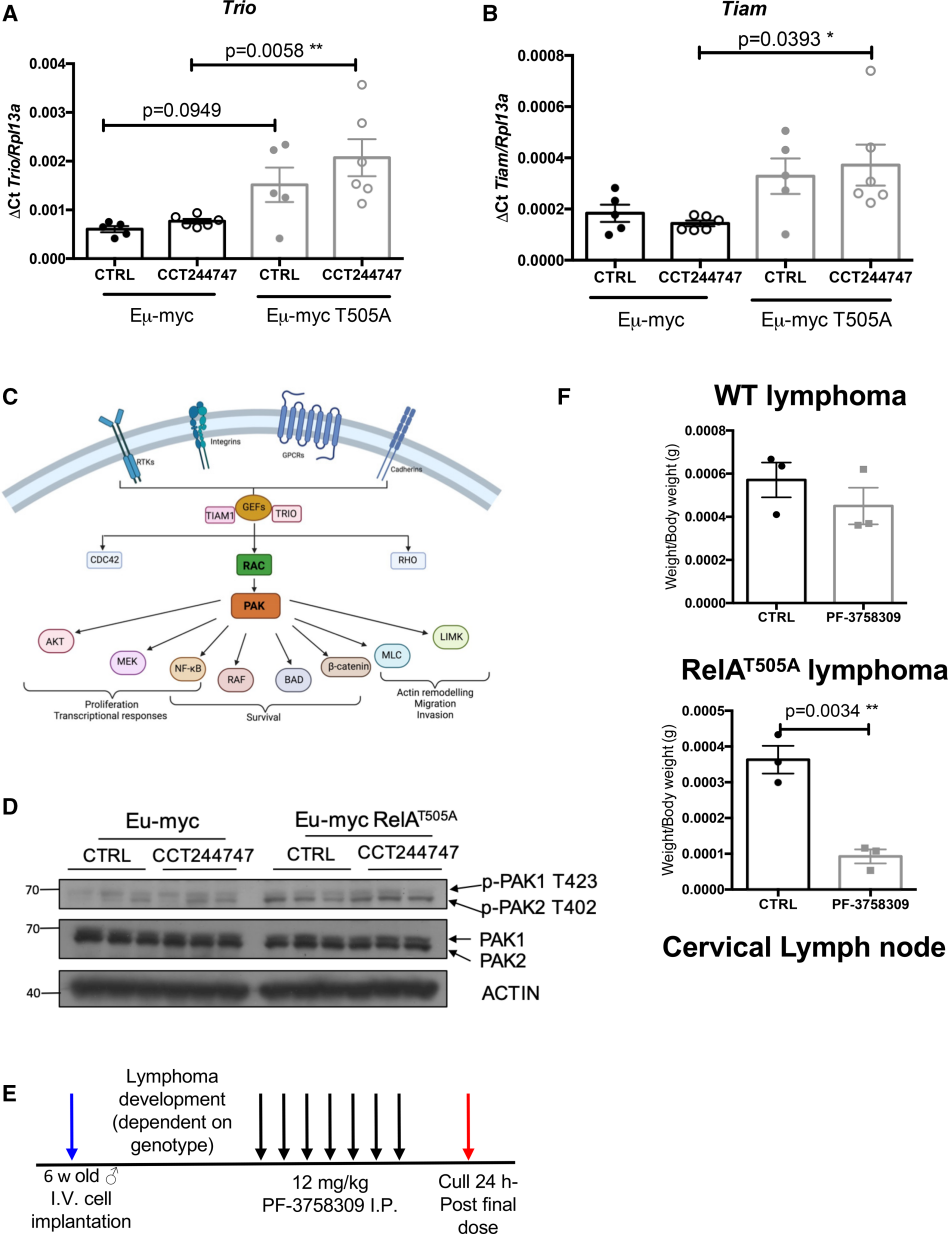
Up-regulation of PAK2 activity in Eµ-Myc/RelA^T505A^ lymphoma cells. (**A** and **B**) Q-PCR validation of RNA-Seq analysis. Relative *Trio* (D) and *Tiam1* (E) transcript levels are significantly up-regulated in tumours from Eµ-Myc/RelA^T505A^ (*n* = 6) when compared with Eµ-Myc WTs (*n* = 5). Data represents mean ± SEM. * *P* < 0.05, ** *P* < 0.01, (One-way ANOVA with multiple comparison analysis). Data represents mean ± SEM, each point is an individual mouse. (**C**) Schematic showing the CDC42, RAC, RHO signalling through the p21-activated kinases (PAK) family and downstream cellular processes mediated via this cellular transduction pathway. Created with BioRender.com. (**D**) Western blot analysis of phospho-PAK1/2 (T423/T402), PAK1/2 or actin in snap frozen tumour extracts prepared from re-implanted Eµ-Myc and Eµ-Myc/RelA^T505A^ tumours from inguinal lymph nodes 8 h following a single dose of CCT244747. The data shows that the signalling through PAK2 is highly active in the Eµ-Myc/RelA^T505A^ cells. (**E**) Schematic diagram illustrating the PI3Ki *in vivo* study in Eµ-Myc and Eµ-Myc/RelA^T505A^ mice. Six week-old C57Bl/6 WT mice were implanted with either Eµ-Myc or Eµ-Myc/RelA^T505A^ (blue arrow) and once tumours became palpable were treated with either 12 mg/kg PF-3758309 i.p. or vehicle control once daily for 7 days (black arrows). Mice were euthanised 24 h after the final dose (red arrow) and tumour burden assessed. (**F**) Scatter plot showing the response of reimplanted Eµ-Myc and Eµ-Myc/RelA^T505A^ tumours to PF-3758309 in the cervical lymph node tumour site. Each tumour was implanted into six syngeneic recipient C57Bl/6 mice, three were treated with PF-3758309 (12 mg/kg i.p.), and 3 with vehicle control, for 7 days once tumours became palpable. A response was defined as a significant reduction (or increase) in tumour burden (*P* < 0.05) using unpaired Student's *t*-tests. WT Eµ-Myc showed little response to PF-3758309 whereas the Eµ-Myc/RelA^T505A^ tumours were reduced by PF-3758309.

It seemed likely therefore that the components of any pathways that might be up-regulated in Eµ-Myc/RelA^T505A^ lymphoma cells fell below the threshold of detectability for the proteomics analysis. This may reflect our observation elsewhere that unlike the Eµ-Myc/Rel^−/−^ lymphoma cells, Eµ-Myc/RelA^T505A^ lymphoma cells retain CHK1 protein [18] and so any effects in CHK1i untreated cells may be more subtle. Therefore, we used our RNA-Seq dataset (Supplementary Data Files S5, S6) as a means to identify up-regulated compensatory bypass signalling pathways in the Eµ-Myc/*RelA^T505A^* cells. Here, we focussed on intrinsic differences in gene expression profiles encoding cell signalling pathways in the re-implanted WT and Eµ-Myc/*RelA^T505A^* lymphomas in the absence of CCT244747 treatment. Functional profiling of the genes whose mRNA expression varied between WT and Eµ-Myc/*RelA^T505A^* cells, revealed an up-regulation of small GTPase transduction pathways, particularly those signalling through RHOA and RAC1 (Figure 4A–C, Supplementary Figure S8B). This included transcript levels of two guanine nucleotide exchange factors (GEFs), TRIO and TIAM1 [47–49], which we subsequently validated as being up-regulated in Eµ-Myc/*RelA^T505A^* lymphomas using qPCR (Figure 5A,B). Interestingly, these genes are also significantly up-regulated in Eµ-Myc/*cRel*^−/−^ lymphomas (S8C & D). TRIO is a GEF for both RHOA and RAC1, whilst TIAM1 regulates RAC1 signalling [50–52]. Both are known to play important roles in cell invasion, metastasis and actin cytoskeleton formation [50–52], all of which we have previously shown to be mediated by phosphorylation of RelA on T505 [13,18,53].

Neither manual examination, STRING analysis nor functional annotation clustering using our phosphoproteomic data gave any further insights into whether RHOA or RAC1 signalling might be up-regulated in Eµ-Myc/*RelA^T505A^* lymphomas (not shown). Therefore, to determine if these changes in transcript level of key RHOA/RAC1 signal transducers resulted in downstream biochemical effects, we took a candidate approach to try and identify potential effectors of this pathway. We thus examined the phosphorylation status of the p21-activated kinases (PAKs) 1 and 2, as these are known effectors of RAC signalling [54] (Supplementary Figure S6C). Phosphorylation of PAK2 on T402 is critical for its activation [55], and is known to control cell growth and survival in cancer [56]. PAK2 is known to directly phosphorylate a number of downstream targets including MAPK4 and MAPK6, leading to the control of cell migration and F-actin polymerisation [57,58]. Western blotting revealed no significant difference in PAK1/2 protein levels but did show marked elevation of PAK2 T402 phosphorylation in the RelA^T505A^ lymphomas (Figure 5D, Supplementary Figure S9A). In agreement, no significant change in PAK2 protein levels were observed in the proteomics dataset in the Eµ-Myc/*RelA^T505A^* lymphomas compared with WT cells; no phosphopeptides were observed for the region surrounding T402 (Supplementary Data File S1). Enhanced activating phosphorylation appeared specific for PAK2, with little change being apparent for PAK1 phosphorylation at T423 (Figure 5D, Supplementary Figure S9A). PAK2 phosphorylation and total protein levels were also up-regulated in Eµ-Myc/*cRel*^−/−^ lymphoma cells (Supplementary Figure S9B).

### Inhibition of PAK signalling provides an alternative therapeutic strategy in Eµ-Myc/RelA^T505A^ lymphomas

Our findings showing up-regulation of RHOA/RAC/PAK signalling in Eµ-Myc/RelA^T505A^ lymphomas, suggests that this pathway may function as a potential mechanism of CHK1i resistance bypass signalling. We therefore decided to evaluate the effectiveness of a PAK inhibitor, PF-3758309 which has already been reported to be efficacious in models of T-cell leukaemia/lymphoma [59] *in vivo,* by analysing its effect on the growth of three transplanted WT Eµ-Myc and Eµ-Myc/RelA^T505A^ tumours. As previously performed with CCT244747 [18,20], each tumour was implanted into six syngeneic C57Bl/6 recipient mice and three were treated intraperitoneally with PF-3758309 once a day for seven days, while three received a vehicle control (Figure 5E). After treatment, we observed a striking reduction in lymphoid tumour burden in all mice re-implanted with Eµ-Myc/RelA^T505A^ tumours and treated with PF-3758309 (Figure 5F, Supplementary Figure S9C,D). In contrast, in all of the WT Eµ-Myc tumours, no significant reduction in lymphoid tumour burden was seen after PF-3758309 treatment. These data suggested that RHOA/RAC/PAK signalling compensates for alterations in CHK1 activity in Eµ-Myc/RelA^T505A^ lymphomas. As with PI3K/AKT signalling above, targeting this pathway may also represent a viable therapeutic strategy for the treatment of CHK1i resistant tumours in human patients.

## Discussion

Resistance to kinase inhibitors can develop via a variety of different mechanisms, such as mutation of the drug target or a gene within an associated signalling network and this presents a new clinical challenge in the development of personalised medicine for cancer therapeutics [21]. It is highly likely that the use of CHK1 inhibitors in the clinic will encounter these same issues. We have developed and characterised two mouse Eµ-Myc B-cell lymphoma models of *de novo* CHK1i resistance, arising from mutations in the c-Rel and RelA NF-κB subunits, alongside cell line models of acquired CHK1i resistance, which underline the critical role that intrinsic target and/or pathway rewiring plays in this context [18,20]. Here, we have used these Eµ-Myc models to identify two compensatory bypass signalling pathways used by Eµ-Myc lymphomas for continued growth, proliferation and survival in the absence of optimal ATR–CHK1 signalling.

Proteomic data from our laboratory demonstrated that Eµ-Myc/*c-Rel*^−/−^ lymphomas strikingly resemble wild type Eµ-Myc cells that had received the CHK1 inhibitor [20]. Further investigation demonstrated that this resulted from almost total loss of CHK1 protein in the Eµ-Myc/*c-Rel*^−/−^ lymphomas [20] (Supplementary Figure S1A). This raised the fundamental question of how these lymphoma cells were still able to survive, given that CHK1 is required in the WT lymphomas to cope with high levels of MYC-induced DNA replication stress (Supplementary Figure S1B). However, we had also observed significant up-regulation of phosphopeptide and total protein levels in both WT Eµ-Myc lymphomas treated with the CHK1 inhibitor, as well as intrinsically in Eµ-Myc/*c-Rel*^−/−^ cells ([20] and Figure 2). These findings suggested that activation of compensatory signalling pathways was allowing the Eµ-Myc/*c-Rel*^−/−^ lymphomas to survive and proliferate, even in the absence of CHK1 [20].

Further examination of the phosphoproteomic data and subsequent western blot analysis of protein extracts, revealed that PI3K/AKT pathway activity was up-regulated in Eµ-Myc/*c-Rel*^−/−^ lymphomas (Figures 2, 3). Moreover, treatment with the PI3K inhibitor GDC-0941/Pictilisib, selectively targeted the Eµ-Myc/*c-Rel*^−/−^ lymphomas, while having no effect on their WT equivalents (Figure 3, Supplementary Figure S7). We also found that Eµ-Myc/RelA^T505A^ lymphomas were sensitive to GDC-0941/Pictilisib treatment (Figure 3, Supplementary Figure S7). These cells do not exhibit the total loss of CHK1 protein we observed in Eµ-Myc/*c-Rel*^−/−^ lymphomas, instead displaying a reduced and altered response to CHK1 inhibition [18]. We also did not see the super induction of PI3K/AKT activity found in the Eµ-Myc/*c-Rel*^−/−^ lymphomas, although western blot data did show that this pathway is active in both the WT and Eµ-Myc/RelA^T505A^ cells (Figure 3, Supplementary Figure S6).

In this report we have not defined the mechanism that drives up-regulation of PI3K/AKT signalling in Eµ-Myc/*c-Rel*^−/−^ lymphomas, although one possibility is that this results from the loss of CHK1 protein that we see in Eµ-Myc/*c-Rel*^−/−^ lymphomas [20]. Our phosphoproteomic data from WT Eµ-Myc lymphomas treated with a single dose of CCT244747 revealed significant overlap of up-regulated phosphopeptides with those seen in Eµ-Myc/*c-Rel*^−/−^ lymphomas (Figure 1). This suggests that inhibiting CHK1 results in the rewiring of other cell signalling pathways. Indeed cross-talk between these pathways has been previously described with, for example, AKT being reported to phosphorylate CHK1 at S280, leading to its inactivation [60]. However, there are other explanations that may either contribute towards this effect or be the primary reason for the induction of PI3K/AKT activity we observe in in Eµ-Myc/*c-Rel*^−/−^ lymphomas. For example, we have identified down-regulation of USP1 mRNA and protein in the Eµ-Myc/*c-Rel*^−/−^ lymphomas [20]. USP1 is a deubiquitinase (DUB) and regulator of DNA replication stress [61–64] and we propose that loss of USP1 results in degradation of CHK1 through the ubiquitin proteasome pathway [20]. USP1 has also previously been found to be a negative regulator of AKT pathway signalling and to target AKT directly [65–68]. Therefore, loss of USP1 in the Eµ-Myc/*c-Rel*^−/−^ lymphomas might act as signalling pathway switch where CHK1 protein is lost concomitant with PI3K/AKT activity being up-regulated. Furthermore, loss of c-Rel expression may have other effects that contribute to the alterations to signalling pathways we observe. An important future area for investigation will be to develop an Eµ-Myc model of acquired CHK1i resistance by reimplanting WT Eμ-Myc lymphoma cells and subjecting them to multiple rounds of CCT244747 treatment and recovery. It would then be possible to determine to what extent these Eμ-Myc lymphoma cells resemble either the Eµ-Myc/*c*-*Rel*^−/−^ or Eµ-Myc/*RelA^T505A^* lymphomas.

Through analysing our RNA-Seq dataset for the presence of intrinsically up-regulated gene expression signatures in Eµ-Myc/RelA^T505A^ lymphomas, we discovered elevated expression of genes encoding components of the RAC1/RHOA signalling pathway, something also seen in Eµ-Myc/*c-Rel*^−/−^ lymphomas (Figure 4, Supplementary Figure S8). This subsequently led to the identification of enhanced levels of activity of the RAC pathway effector PAK2 activity in both the Eµ-Myc/RelA^T505A^ and Eµ-Myc/*c-Rel*^−/−^ lymphomas. Moreover, selective targeting of Eµ-Myc/RelA^T505A^ (but not WT) lymphomas with the PAK inhibitor PF-3758309 (Figure 5, Supplementary Figure S9), identified this as another potential bypass pathway activated in response to CHK1i resistance. However, similar to the situation with the PI3K/AKT pathway discussed above, we have not identified the mechanism that underlies this finding. Further experimentation will be required using models of acquired CHK1i resistance to determine whether this is a common feature of CHK1i resistance or a consequence of disrupted NF-κB signalling in the context of the Eµ-Myc lymphoma model. Nonetheless, targeting PAK2 could, similar to targeting PI3K/AKT activity, represent a future strategy for the treatment of patients with cancers that have acquired CHK1i resistance.

**Figure 6.**
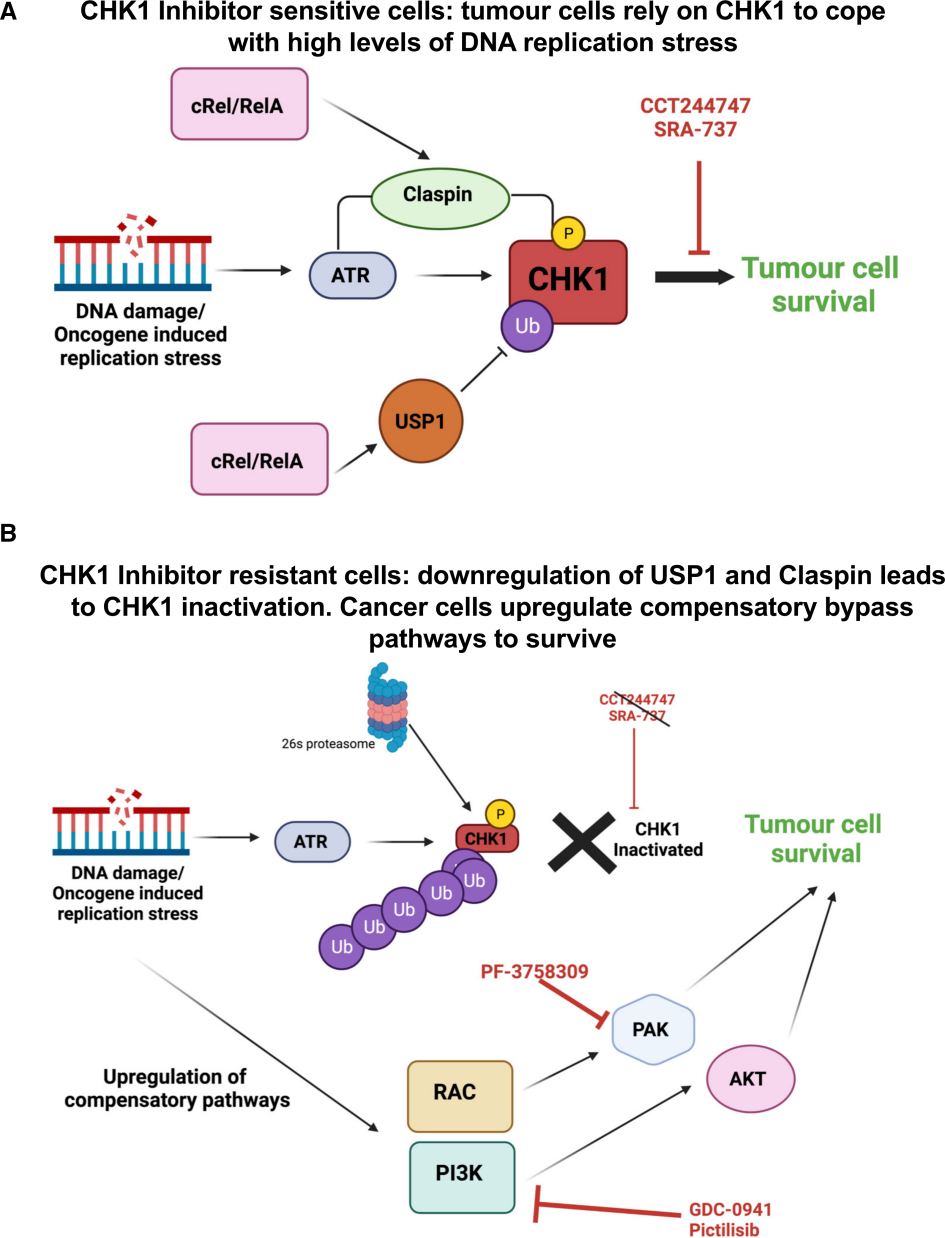
Schematic model summarising the development of CHK1i resistance and activation of bypass pathway signalling in Eµ-Myc/*c-Rel*^−/−^ and Eµ-Myc/RelA^T505A^ lymphomas. (**A**) MYC-driven tumours, or those undergoing high levels of replication stress become addicted to ATR–CHK1 signalling. The NF-κB subunits c-Rel or RelA can induce the levels of the adaptor protein Claspin, which facilitates the phosphorylation of CHK1 by ATR, together with USP1, a CHK1 deubiquitinase. Together this has the effect of maintaining high levels of CHK1 activity, required by the cancer cell. Consequently, these cells are sensitive to CHK1 inhibition by CCT244747 or SRA737, leading to tumour cell death. (**B**) Our data indicates that both *de novo* and acquired resistance to CHK1i can require two events. In the first of these CHK1 protein or activity is lost, which can result from down-regulation of Claspin or USP1 expression. The loss of CHK1 activity removes the target of the CHK1 inhibitor. However, to survive loss of CHK1 activity, the tumour cells need to up-regulate compensatory bypass pathways. In our models we observed the activation of the PI3K/AKT and RAC/RHO/PAK2 pathways in CHK1i resistant cells. These CHK1 inhibitor resistant cells can then be targeted with agents such as the PI3K inhibitor, GDC-0941/Pictilisib or the PAK inhibitor, PF3758309, leading to tumour cell death.

Taken together, we propose that our data reveals that in the Eµ-Myc model, CHK1 inhibitor resistance can develop through a two-step process. The first of these is the loss or alteration of CHK1 signalling mediated, at least in part, by loss of USP1 [20] or Claspin expression [18] (Figure 6). To enable the lymphoma cells to survive loss of CHK1, which they had been addicted to as a means of surviving high levels of MYC-induced DNA replication stress, they simultaneously activate a network of parallel survival pathways, including PI3K/AKT and RAC/PAK. Whether these effects are a consequence of single event, such as down-regulation of USP1 [20], or whether there are other as yet uncharacterised effects of mutating NF-κB subunits, remains to be determined. Other mechanisms of CHK1 inhibitor resistance have been reported [69–72]. It is likely therefore that the route a cancer cell can take to achieve this, will depend on the nature of its driver mutations and tissue type. Further work is needed to determine how widespread the model we have proposed here is in human cancers. Nonetheless, our data implies that at least for some tumour types such as those driven by MYC amplification, co-targeting of PI3K, AKT and/or PAK will increase the clinical efficiency and success of CHK1 inhibition. Alternatively, inhibiting these pathways may represent a second line of therapy where tumours have developed resistance to drugs targeting checkpoint kinases.

## Methods

### Ethics statement

All mouse experiments were approved by Newcastle University's Animal Welfare and Ethical Review Board. All procedures, including the of breeding genetically modified mice, were carried out under project and personal licenses approved by the Secretary of State for the Home Office, under the United Kingdom's 1986 Animal (Scientific Procedures).

### Drugs and compounds

GDC-0941/Pictilisib (HY-50094) and PF-3758309 (HY-13007) were purchased from Med Chem Express. All other compounds were purchased from Sigma–Aldrich.

### Reimplantation studies

For tumour therapy studies, 2 × 10^6^ Eµ-Myc, Eµ-Myc*/c-Rel*^−/−^or Eµ-Myc/RelA^T505A^ tumour cells from male mice were transplanted intravenously (IV) via the lateral tail vein into 8-week old male C57BL/6 recipients. Mice were monitored daily using parameters such as their bodyweight and food and water consumption to assess disease progression. Mice were necropsied when they became moribund and the tumour burden assessed. Mice were humanely sacrificed by cervical dislocation. No anaesthesia was used at any point during any studies described here.

Oral administration of the PI3K inhibitor, GDC-0941, prepared as previously described [74], or vehicle control (0.5% methyl cellulose (M7140), 0.2% Tween-80 (P4780) (Sigma–Aldrich) was initiated when tumours became palpable (∼10 days after inoculation of Eµ-Myc or Eµ-Myc/RelA^T505A^ cells, and 20 days after inoculation of Eµ-Myc*/c-Rel*^−/−^ cells) and given as a single agent, bolus dose (100 mg/kg p.o.) for nine consecutive days. Lymphoid tumour burden and final tumour weights were measured at necropsy 24 h after the final dose.

Intra-peritoneal administration of the PAK1/2 inhibitor, PF-3758309, prepared as previously described [59], or vehicle control (2% carboxymethyl cellulose (419273) (Sigma–Aldrich)) was initiated when tumours became palpable and given as a single agent, bolus dose (12 mg/kg p.o.) for seven consecutive days. Lymphoid tumour burden and final tumour weights were measured at necropsy 24 h after the final dose.

### Gene expression analysis using quantitative real-time PCR

Total RNA was puriﬁed from snap frozen tumour tissue from Eµ-Myc or Eµ-Myc/*c-Rel*^−/−^ or Eµ-Myc*/RelA^T505A^* mice following homogenisation using Precellys 24 ceramic mix bead tubes (431-0170, Stretton Scientific Ltd) in a Precellys 24 benchtop homogeniser (Stretton Scientific Ltd) at 6500 rpm for 30 s. After this, samples were passed through Qiashredders (79656, Qiagen, Crawley, U.K.) and RNA was purified using the Qiagen RNeasy mini kit (74004) according to manufacturer's instructions.

RNA was measured for purity and concentration with the NanoDrop1000 (ThermoFisher Scientific) and reverse transcribed using the Quantitect Reverse transcription Kit (205311, Qiagen) according to manufacturer's instructions. Quantitative real-time PCR was performed on 20 ng cDNA, in triplicate, using predesigned Quanititect Primer assays (Qiagen) to the following murine genes; *Trio* (QT01198960)*, Tiam1*(QT00126819)*.* These samples were run and analysed on a Rotor-gene Q system (Qiagen), using murine *Rpl13a* primers (QT00267197) as an internal control. All CT values were normalised to *Rpl13a* levels as indicated in the figures.

### Western blotting

Whole cell extracts were prepared from Eµ-Myc, Eµ-Myc/*c-Rel*^−/−^ or Eµ-Myc*/RelA^T505A^*extracted direct from snap frozen pieces of tumour. These were lysed in PhosphoSafe™ Extraction Reagent using the Precellys24 ceramic mix bead tubes (Stretton Scientific Ltd) in a Precellys®24 homogeniser (Stretton Scientific Ltd) at 6500 rpm for 30 s, then extracted according to the PhosphoSafe™ Extraction Reagent manufacturer's instructions. Protein quantification was undertaken using the BCA protein assay (23225, Thermo-scientific, U.K.), and samples resolved by standard denaturing SDS–PAGE gels (3450034, Bio-Rad, U.K.). Samples were transferred onto PVDF membrane (GVWP04700 Merck-Millipore) before being probed with the primary antibody. Horseradish peroxidase-conjugated secondary antibodies and enhanced chemiluminscence reagent (34076 Thermo-scientific, U.K.) were used for detection.

### Antibodies

Antibodies used were GSK3B (phospho S9) (9336 Cell Signalling), GSK3B (9315 Cell Signalling), ERK 1/2 (phospho T202, Y204) (9101 Cell Signalling), ERK 1/2 (9102 Cell Signalling), AKT (phospho S473) (9271 Cell Signalling), AKT (phospho T308) (9275 Cell Signalling), AKT (9272 Cell Signalling), JNK (phospho T183, Y195) (9251 Cell Signalling), JNK (9252 Cell Signalling), p38 (phospho T180, Y182) (9211 Cell Signalling), p38 (9212 Cell Signalling), B-ACTIN (A5441 Sigma), PEA15 (2780 Cell Signalling), PI3K (phospho Y458) (4228 Cell Signalling), PI3K (4229 Cell Signalling). PAK1/2 (phospho T423/T402) (2605 Cell Signalling), PAK1/2 (2604 Cell Signalling), Anti-rabbit IgG (A6154 Sigma and 7074 Cell Signalling) and anti-mouse IgG (7076 Cell Signalling) HRP-linked secondary antibodies were used for western blot detection.

### Proteomics and analysis

Tissue extracts were prepared from snap frozen pieces of Eµ-Myc, Eµ-Myc/c-Rel^−/−^ Eµ-Myc/RelA^T505A^ or splenic tumours. Briefly, tissue samples were suspended in 100 mM triethylammoniumbicarbonate (TEAB) with a mixture of protease and phosphatase inhibitors (cOmplete Mini EDTA-free protease inhibitor cocktail plus PhoSTOP phosphatase inhibitor cocktail, both obtained from Roche), homogenised by bead beater, and sonicated on ice. Lysed extracts were incubated with 0.1% (w/v) Rapigest SF (Waters) for 10 min at 80°C, left to cool, and incubated for 10 min on ice with Benzonase endonuclease (Merck Millipore) to digest nucleic acids. Samples were centrifuged (14 000***g***, 10 min at 4°C) to pellet cell debris. Protein concentration of the clarified lysate was ascertained by Bradford assay. Protein (200 µg) from each sample was aliquoted for protein digestion.

Disulfide bonds were reduced (4 mM DTT in 100 mM TEAB, 10 min at 60°C) and free cysteines alkylated with iodoacetamide (14 mM in 100 mM TEAB, for 30 min, RT in the dark). Iodoacetamide was quenched by addition of DTT to a final concentration of 7 mM. Proteins were digested with 2% (w/w) trypsin overnight at 37°C with gentle agitation. Resultant peptides were labelled with TMT 6-plex reagents (Thermo Scientific) at an 8 : 1 tag:protein ratio as per the manufacturer's instructions, with labels assigned to samples randomly for the first biological replicate and shifted for each subsequent replicate. The labelling reaction was quenched by addition of 0.3% (v/v) hydroxylamine (Thermo Scientific) in 100 mM TEAB. TMT labelled peptides were mixed and dried to completion by vacuum centrifugation before re-suspending in 100 mM TEAB/ 1% TFA to hydrolyse the Rapigest SF (RT, 10 min). Insoluble Rapigest SF cleavage product was removed by centrifugation (13 000***g*** for 15 min at 4°C), and the sample desalted using C18 spin columns (Pierce, #89852) as per the manufacturers protocol, prior to strong cation exchange using stage tips (packed in-house with 5 disks per 200 µl tip as described previously [75] (Empore Supelco 47 mm Cation Exchange disk, #2251)). Each mixed labelled peptide sample was split across 8 tips, with peptides passed through the equilibrated stage tips twice. Bound peptides were eluted with 5% NH_4_OH (3 × 100 µl) and dried to completion using a vacuum centrifuge.

Peptides were fractionated using basic reverse-phase liquid chromatography as described, with 65 fractions collected, partially dried by vacuum centrifugation, and concatenated into five pools. For each pool, 5% was aliquoted and dried to completion prior to MS analysis. The remaining 95% was subjected to TiO_2_-based phosphopeptide enrichment, as described previously.

Total protein and phosphopeptide enriched fractions were analysed by LC–MS/MS using an UltiMate 3000 RSLCTM nano system (Dionex) coupled in-line with a Thermo Orbitrap Fusion Tribrid mass spectrometer (Thermo Scientific). Peptides were loaded onto the trapping column (PepMap100, C18, 300 µm × 5 mm, Thermo Scientific) using partial loop injection with 2% acetonitrile (ACN), 0.1% TFA at a flow rate of 9 µl/min for 7 min. Peptides were resolved on an analytical column (Easy-Spray C18, 75 µm × 500 mm, 2 µm bead diameter) using a gradient from 96.2% A (0.1% formic acid):3.8% B (80% ACN, 0.1% formic acid) to 50% B over either 120 min (single injection for phosphopeptide-enriched samples and two injections for total protein samples) or 240 min (single injection for total protein samples only) at a flow rate of 300 nl/min. Full MS1 spectra were acquired in the Orbitrap over *m/z* 375-2000 (60K resolution at m/z 200), with a maximum injection time of 50 ms and an ACG target of 4 × 10^5^ ions. Data-dependent MS2 analysis was performed using a top speed approach (3 s cycle time) with peptides fragmented by collision-induced dissociation [76] at a normalised collision energy [77] of 35%, with fragment ions detected in the ion trap (maximum injection time of 50 ms, ACG target of 1 × 10^4^). Following acquisition of each MS2 spectrum, a synchronous precursor selection (SPS) MS3 scan was performed on the top 10 most intense fragment ions, with SPS-MS3 precursors fragmented using higher energy collision-induced dissociation (HCD), at an NCE of 65%, and analysed using the Orbitrap over *m/z* 100–500 (50K resolution at m/z 200) with a maximum injection time of 105 ms and an ACG target of 1 × 10^5^ [78,79].

Analysis of MS data, with quantification of TMT reporter ion distributions, was performed using Proteome Discoverer 2.4 (PD 2.4) in conjunction with MASCOT (v2.6) and Percolator. For peptide identification from MS2 spectra, raw data files were converted to mzML format and searched in MASCOT against the Mouse UniProt reviewed database (Downloaded 25/04/2018; 16 966 sequences) with parameters set as follows: MS1 tolerance of 10 ppm; MS2 tolerance of 0.6 Da; enzyme specificity was set as trypsin with two missed cleavages allowed; carbamidomethylation of cysteine and TMT 6-plex modifications (on peptide N-termini and lysine side chains) were set as fixed modifications; oxidation of methionine and acetylation of protein N-termini were set as variable modifications, with the addition of phosphorylation (at serine, threonine or tyrosine residues) for phosphopeptide-enriched samples. Percolator was used for control of false discovery rates with a target FDR of 0.05. For phosphopeptide-enriched samples, the ptmRS node, operated in phosphoRS mode, was added to the PD 2.4 workflow for phosphosite localisation. The ptmRS node in Proteome Discoverer is a modified version of phosphoRS [80] that provides a confidence localisation score for post-translational modifications (PTMs), in this case phosphorylation [81]. In parallel with peptide identification, relative quantification of TMT 6-plex reporter ions was performed in PD 2.4 using the ‘Reporter ions quantifier’ node, to quantify reporter ions from MS3 spectra with a peak integration tolerance of 20 ppm using the ‘most confident centroid’ integration method. Normalisation to total peptide amount was performed within PD 2.4, with peptide group abundances summed for each sample and a normalisation factor calculated from the sum of each sample and the maximum sum in all files.

Quantitative ratios were calculated for each biological replicate to look for protein/phosphopeptide changes. Quantitative ratios were log_2_ transformed and, for all proteins/phosphopeptides quantified in at least three out of five bioreps, statistical analysis was performed in R using the LIMMA package, using a *P* ≤ 0.05 significance cut off. Pearson correlation analysis was performed in R using the ggscatter package, with a linear regression line and 95% confidence intervals included on each plot. The mass spectrometry proteomics data have been deposited to the ProteomeXchange Consortium (http://proteomecentral.proteomexchange.org) via the PRIDE partner repository with the dataset identifiers Project accession: PXD026203 & Project DOI: 10.6019/PXD026203. Please note that data from this proteomics study is also included in our analysis Eµ-Myc/c-Rel^−/−^ and Eµ-Myc/RelA^T505A^ lymphomas described elsewhere [18,20]. Consequently, this description of the methods is duplicated in those papers. We have compiled supplementary data from proteomics analysis in the study into a single file (Supplementary Data File S1), which is also attached to the other papers that analyse this data [18,20].

### RNA-Seq and analysis

RNA was extracted as described above and sample quality analysed using Tapestation automated electrophoresis (Aglient) according to manufacturer's instructions. Sample RNA Integrity Number (RIN) score exceeded six in all cases. mRNA-Seq libraries were prepared using the Illumina TruSeq Stranded mRNA kit following manufacturer's reference guide and sequenced on an Illumina NextSeq 500 high-output 75 cycle flow cell, generating 25 million 75 bp single reads per sample. The raw sequence data quality was first inspected using FastQC and MultiQC. Transcript counts were generated via Salmon [82] using Release M20 (GRCm38.p6) of the mouse genome (for the mouse samples) and Release 31 (GRCh38.p12) of the human genome (for the human samples).

The quantification files were imported into R for gene-level analyses using tximport and the differential gene expression analyses were carried out using DESeq2. The data has been deposited on ENA (https://www.ebi.ac.uk/ena/submit/sra/#home) with the accession number PRJEB45284.

Please note that data from this RNASeq analysis is also used in the analysis of changes in Eµ-Myc/c-Rel^−/−^ and Eµ-Myc/RelA^T505A^ lymphomas described elsewhere [18,20]. Consequently, this description of the methods is duplicated in these papers. We have compiled supplementary data from RNA Seq analysis in the study into single files (Supplementary Data Files S4, S5), which are also attached to the other papers that analyse this data [18,20].

### STRING and Venn diagram analysis

STRING analysis was performed using version 11.0 at https://string-db.org/ [40]. Where indicated AKT1, ERK1 (MAPK3) or JNK1 (MAPK8) were manually added to the protein list to determine connections to phosphoproteins identified from the proteomics analysis. Analysis of connections was performed under medium or high confidence settings as described in figure legends, using homo sapiens as the species setting. All sources of evidence were applied to the analysis (Textmining, Experiments, Databases, Co-expression, Neighbourhood, Gene Fusion & Co-occurrence). Connections were limited to query proteins only. In all STRING analysis shown, the lines connecting proteins indicate both functional and physical associations with the line thickness indicates the strength of data support. Details on proteins analysed and connections are in Supplementary Data File S4.

Venn diagram analysis was performed at http://bioinformatics.psb.ugent.be/webtools/Venn/ with figures being created using https://www.biovenn.nl/index.php. Details of Venn diagram results are in Supplementary Data File S3.

### Statistical analysis

GraphPad Prism software (http://www.graphpad.com, V6.0) was used for statistical analysis. Except where stated in figure legends, unpaired *t*-tests or One-way ANOVA were used to calculate *P* values (*P* values of *P* < 0.05 were considered signiﬁcant). Pearson correlations were performed using the ggscatter package in R, with linear regression lines fitted with 95% confidence intervals.

## Data Availability

The mass spectrometry proteomics data have been deposited to the ProteomeXchange Consortium (http://proteomecentral.proteomexchange.org) via the PRIDE partner repository [73] with the dataset identifiers Project accession: PXD026203 & Project DOI: 10.6019/PXD026203. RNASeq data has been deposited on ENA (https://www.ebi.ac.uk/ena/submit/sra/#home) with the accession number PRJEB45284. The authors are happy to provide all original data, and for this to be shared on Figshare as appropriate.

## Open Access

Open access for this article was enabled by the participation of the University of Liverpool in an all-inclusive *Read & Publish* agreement with Portland Press and the Biochemical Society under a transformative agreement with JISC.

## Abbreviations

ACN: acetonitrile
ATR: Ataxia Telangiectasia and Rad3 Related
CHK1i: CHK1 inhibitors
FDR: false discovery rate
MAPK: MAP kinase
PAKs: p21-activated kinases
PTMs: post-translational modifications
SPS: synchronous precursor selection
TEAB: triethylammoniumbicarbonate
TMT: tandem mass tag
