# Supplementary material for: Up-regulation of the PI3K/AKT and RHO/RAC/PAK signalling pathways in CHK1 inhibitor resistant Eµ-Myc lymphoma cells
Source: Biochem J. 2022 Oct 14;479(19):2131–51. doi: 10.1042/BCJ20220103 (PMC9704644; doi:10.1042/BCJ20220103)
Supplement: Supplementary Material [file BCJ-479-2131-s1.pdf]

Supplementary Information

Upregulation of the PI3K/AKT and RHO/RAC/PAK signalling pathways in CHK1 inhibitor resistant Eμ-Myc lymphoma cells

Jill E. Hunter[1], Amy E. Campbell[2], Scott Kerridge[1], Callum Fraser[1], Nicola L. Hannaway[1], Saimir Luli[3], Iglika Ivanova[1], Philip J. Brownridge[2], Jonathan Coxhead[1], Leigh Taylor[1], Peter Leary[4], Megan S.R. Hasoon[4] Claire E. Eyers[2*] and Neil D. Perkins[1*]

[1] Newcastle University Biosciences Institute
Wolfson Childhood Cancer Research Centre
Level 6, Herschel Building
Newcastle University
Brewery Lane
Newcastle upon Tyne, NE1 7RU, UK
[2]Centre for Proteome Research, Department of Biochemistry and Systems Biology,
Institute of Systems, Molecular and Integrative Biology,
University of Liverpool,
Liverpool L69 7ZB, U.K.
[3]Newcastle University Clinical and Translational Research Institute
Preclinical In Vivo Imaging (PIVI)
Faculty of Medical Sciences
Newcastle University
Newcastle Upon Tyne, NE2 4HH, UK
[4]Bioinformatics Support Unit,
Faculty of Medical Sciences
Newcastle University
Newcastle Upon Tyne, NE2 4HH, UK

* corresponding authors

Tel. 0191 2088866 Email: neil.perkins@ncl.ac.uk

Tel. 0151 794424 Email: Claire.Eyers@liverpool.ac.uk

Supplementary Figure Legends

Figure S1. Work leading up this study and the key unanswered question

(A) Summary of the work leading up to this study that characterised changes to CHK1 protein and activity in CCT244747 resistant REL-/- and RELA^{T505A} Eμ-Myc lymphoma cells. In REL-/- Eμ-Myc lymphoma cells, CHK1 protein is lost and consistent with this, the phospho and total proteomic signature in the absence of any drug treatment shows a high degree of overlap with that seen in WT Eμ-Myc lymphoma cells that have been treated with the CHK1 inhibitor (CHK1i) CCT244747 [20]. Loss of CHK1 protein in the REL-/- Eμ-Myc lymphoma cells is associated with both down regulation of its mRNA and loss of the deubiquitinase (DUB) USP1 that can act to stabilise CHK1 protein [20]. These cells also lose expression of Claspin, an adaptor protein required for ATR phosphorylation and activation of CHK1 [18]. In RELA^{T505A} Eμ-Myc lymphoma cells, CHK1 protein is still present [18]. However, after CCT244747 treatment the altered phosphopeptide proteomic signature if different to that seen in WT cells. Fewer phosphopeptides are seen to be changed in the RELA^{T505A} Eμ-Myc lymphoma cells, with many of these also being different to those seen in WT cells [18]. Similar to REL-/- Eμ-Myc lymphomas, RELA^{T505A} cells also have lower levels of Claspin (and USP1, albeit to a lesser extent. Potentially accounting for the altered CCT244747 induced phosphopeptide signature between WT and RELA^{T505A} Eμ-Myc lymphoma cells, total proteome data revealed differences in the expression of a number of proteins linked to or known to interact with CHK1 [18]. Consistent with these *in vivo* studies, in U2OS and Huh7 cell line models with CCT244747 resistance we also observed downregulation of CHK1, Claspin and USP1 expression [20].

B) Diagram depicting the aim of this study. WT Eμ-Myc lymphomas, in common with many types of cancer, are dependent upon CHK1 to cope with the high levels of ongoing DNA replication stress. Consequently, treatment with a CHK1 inhibitor (CHK1i) results in tumour cell death due to the accumulation of damaged DNA that ultimately results in genomic catastrophe. In work leading up this study [18, 20], we found that REL-/- and RELA^{T505A} Eμ-

Myc lymphomas have either lost CHK1 or display altered CHK1 activity respectively [18, 20]. A consequence of this is resistance to treatment with the CHK1i CCT244747 [18, 20]. However, despite these changes, which might be expected to have a similar effect to CHK1i treatment and lead to genomic catastrophe, REL-/- and RELA^{T505A} Eμ-Myc lymphomas survive. Indeed, these mice show reduced survival times compared to WT controls [18, 20, 24]. We have therefore investigated what other changes are occurring to signalling pathways in REL-/- and RELA^{T505A} Eμ-Myc lymphomas that promote their continued survival and whether these now represent a vulnerability in cells that have developed CHK1i resistance.

Figure S2. Correlation analysis of upregulated proteins and phosphopeptides. Pearson correlation between fold changes ($\log_2$) for proteins/phosphopeptides up-regulated in Eμ-Myc/c-Rel-/- lymphomas, and fold changes ($\log_2$) for the same proteins/phosphopeptides in WT Eμ-Myc lymphomas after CCT244747 treatment, with both normalised to control treated WT Eμ-Myc lymphomas. Although there is a weak, positive correlation between the changes to both proteins and phosphopeptides from these conditions (Proteins R=0.41, p=1.5E-15, phosphopeptides R=0.29, p=0.00019), the magnitude of the fold change seen in WT Eμ-Myc lymphomas after CCT244747 treatment is generally much less than that seen in Eμ-Myc/c-Rel-/- lymphomas. Blue dots indicated proteins or phosphopeptides that are upregulated in both conditions. Black dots indicate outlier phosphopeptides that are up-regulated in Eμ-Myc/c-Rel-/- lymphomas but downregulated in WT Eμ-Myc lymphomas after CCT244747 treatment. The solid black line represents the linear regression line with the shaded region showing a 95% confidence interval. The dashed line shows where the regression line would fall if fold changes were identical between the compared conditions.

Figure S3. Analysis of potential AKT targets and phosphorylated kinases identified in Eμ-Myc/*c-Rel-/-* lymphoma phosphoproteomic data

(A) Table listing potential AKT targets arising from cross referencing upregulated putative phosphosites identified the Eμ-Myc/*cRel-/-* lymphomas with a list of known AKT target sites

available on the Cell Signalling Technology website (https://www.cellsignal.co.uk/learn-and-support/reference-tables/pi3k-akt-substrates-table). Shown are those sites where the Eμ-Myc/*cRel-/-* phospho site is identical to a previously described AKT target site. Also shown are those phosphosites not shown to be an AKT site but where the target protein has been shown to be phosphorylated by AKT elsewhere. See also Supp Data File 2.

(B) Table listing kinases with upregulated phosphosites in Eμ-Myc/*cRel-/-* lymphomas. See also Supp Data File 2.

Figure S4. Proteins with upregulated phosphopeptides in Eμ-Myc/*cRel-/-* lymphomas functionally linked to AKT1, ERK1, JNK1, and p38 MAPK.

Table listing proteins from STRING analysis of proteins with upregulated phosphopeptides in Eμ-Myc/*cRel-/-* lymphomas relative to WT Eμ-Myc lymphomas that are functionally linked to the kinases AKT1, ERK1 (MAPK3), JNK1 (MAPK8) and p38 MAPK (MAPK14). Analysis was performed under with medium or high STRING confidence settings as shown. Data from this table was used to create images in Fig. 2. See also Supp Data file 4.

Figure S5. **STRING analysis showing the interaction network of proteins with upregulated phosphopeptides in Eμ-Myc/*cRel-/-* lymphomas**

STRING analysis was performed on all proteins with upregulated phosphopeptides in Eμ-Myc/*cRel-/-* lymphomas relative to WT Eμ-Myc lymphomas. The image shown shows the functional links identified with the high confidence STRING setting, with no manual addition of the kinases AKT1, ERK1 and JNK1, and with those proteins that have no links to other proteins in the network removed. Proteins linked to AKT1 in Fig. S3 are highlighted in red. DENND1B, FMNL1 and USP8, which are listed in Fig. S3, are not shown as these are only linked to AKT1 and not the other proteins in this network. See also Supp Data File 4.

Figure S6 Additional western blot analysis of Eμ-Myc protein extracts

(A) Western blot analysis of phospho-Ser473 AKT, AKT, or ACTIN in snap frozen tumour extracts prepared from more re-implanted Eμ-Myc, Eμ-Myc/c-Rel-/- and Eμ-Myc/RelA^{T505A} tumours mouse inguinal lymph nodes 8 hours following a single dose of CCT244747. The data shows that the AKT pathway is highly in Eμ-Myc/c-Rel-/- tumours. Please note that the Actin blot here is different exposure of the one used in Fig S2 (bottom panel) below.

(B) Western blot analysis of phospho-Thr202/Tyr204 ERK1/2, ERK1/2 or ACTIN in snap frozen tumour extracts prepared from two separate sets of mice re-implanted Eμ-Myc, Eμ-Myc/c-Rel-/- and Eμ-Myc/RelA^{T505A} tumours mouse inguinal lymph nodes 8 hours following a single dose of CCT244747. The data shows that the ERK pathway is highly active in Eμ-Myc/c-Rel-/- tumours.

(C) Western blot analysis of phospho-Thr183/Tyr185 JNK1/2, JNK1/2, phospho-Thr180/Tyr182 p38, p38 or ACTIN in snap frozen tumour extracts prepared from two separate sets of mice re-implanted Eμ-Myc, Eμ-Myc/c-Rel-/- tumours and Eμ-Myc/RelA^{T505A} tumours mouse inguinal lymph nodes 8 hours following a single dose of CCT244747. The data shows that the JNK/p38 pathway signalling is reduced in Eμ-Myc/c-Rel-/- tumours. Please note some of the actin blots used here are replicated in another paper (Fig S2C & S6B [20]), where they are used as the controls for USP1 and USP14 expression, also analysed using these membranes. Please note that the Actin blots from upper and lower p38 panels in this figure are also used in another study (Fig. 5C middle panel, Fig S6A lower panel [20], where the same membrane was probed with antibodies to other proteins.

(D) Western blot analysis of PEA15, or ACTIN in snap frozen tumour extracts prepared from re-implanted Eμ-Myc, Eμ-Myc/c-Rel-/- and Eμ-Myc/RelA^{T505A} tumours mouse inguinal lymph nodes 8 hours following a single dose of CCT244747. Please note that the Actin blot used in this figure is the same as that in S5C above (lower JNK panel). They were part of the same original western membrane but now split between 2 figures.

Figure S7. Additional data showing the response of reimplanted Eμ-Myc lymphomas to GDC-0941/Pictilisib

(A) Scatter plot showing the response of two further reimplanted Eμ-Myc, Eμ-Myc/c-Rel-/- and Eμ-Myc/RelA^{T505A} tumours and their response to GDC-0941/Pictilisib in the cervical lymph node tumour sites. Each of the tumours was implanted into 6 syngeneic recipient C57Bl/6 mice, 3 were treated with GDC-0941/Pictilisib (100 mg/kg p.o), and 3 with vehicle control, for 9 days once tumours became palpable. A response was defined as a significant reduction (or increase) in tumour burden ($P<0.05$) using unpaired Student's t-tests. WT Eμ-Myc showed little response to GDC-0941/Pictilisib whereas the Eμ-Myc/c-Rel-/- and Eμ-Myc/RelA^{T505A} tumours were reduced by GDC-0941/Pictilisib.

(B) Table with all lymphoid organ weights in mice that had been implanted with Eμ-Myc, Eμ-Myc/c-Rel-/- or Eμ-Myc/RelA^{T505A} and treated with GDC-0941/Pictilisib or vehicle control. WT Eμ-Myc showed little response to GDC-0941/Pictilisib whereas the Eμ-Myc/c-Rel-/- and Eμ-Myc/RelA^{T505A} tumours were reduced by GDC-0941/Pictilisib.

Fig. S8. Additional analysis of upregulation of RHO/RAC pathway members

(A) Venn diagram depicting the lack of overlap between phosphopeptides upregulated in Eμ-Myc/c-Rel-/- and Eμ-Myc/RelA^{T505A} tumours, relative to WT Eμ-Myc controls.

(B) Bar plot (gene number on x axis, and coloured by adj pval) showing GO enrichment analysis from the Eμ-Myc/RelA^{T505A} RNA-Seq data shown in Fig. 3B.

(C & D) Q-PCR validation of RNA-Seq analysis. Relative *Trio* (B) and *Tiam1* (C) transcript levels are significantly up-regulated in tumours from Eμ-Myc/c-Rel-/- (n=5) when compared with Eμ-Myc WTs (n=5). Data represents mean ± SEM. p* <0.05, p** <0.01, (One-way ANOVA with multiple comparison analysis). Data represents mean ± SEM, each point is an individual mouse.

Fig. S9. Additional data looking at the upregulation and role of PAK2 in Eμ-Myc/RelA^{T505A} lymphomas.

(A) Western blot analysis of phospho-PAK1/2 (T423/T402) or ACTIN in snap frozen tumour extracts prepared from re-implanted Eμ-Myc and Eμ-Myc/RelA^{T505A} tumours from inguinal lymph nodes 8 hours following a single dose of CCT244747. The data shows that the signaling through PAK1/2 pathway is highly active in the Eμ-Myc/RelA^{T505A} cells.

(B) Western blot analysis of phospho-PAK1/2 (T423/T402), PAK1/2 or ACTIN in snap frozen tumour extracts prepared from re-implanted Eμ-Myc and Eμ-Myc/c-Rel-/- tumours from inguinal lymph nodes 8 hours following a single dose of CCT244747. The data shows that the signaling through PAK1/2 pathway is highly active in the Eμ-Myc/c-Rel-/- cells.

(C) Scatter showing the response of two further reimplanted Eμ-Myc and Eμ-Myc/RelA^{T505A} tumours and their response to PF-3758309 in the cervical lymph node tumour site. Each of tumour was implanted into 6 syngeneic recipient C57Bl/6 mice, 3 were treated with PF-3758309 (12 mg/kg i.p.), and 3 with vehicle control, for 7 days once tumours became palpable. A response was defined as a significant reduction (or increase) in tumour burden ($P<0.05$) using unpaired Student's t-tests. WT Eμ-Myc showed little response to PF-3758309 whereas the Eμ-Myc/RelA^{T505A} tumours were reduced by PF-3758309.

(D) Table with all lymphoid organ weights in mice that had been implanted with Eμ-Myc or Eμ-Myc/RelA^{T505A} and treated with PF-3758309 or vehicle control. WT Eμ-Myc showed little response to PF-3758309 whereas the Eμ-Myc/RelA^{T505A} tumours were reduced by PF-3758309.

Supplementary data files

Supp Data File 1 All proteomics data.xlsx

Data from proteomics analysis of reimplanted Eμ-Myc lymphoma cells with either vehicle of CHK1i (CCT244747) treatment for 8 hours. Please note, this data file also accompanies two other manuscripts where we use Eμ-Myc lymphoma cells [18, 20].

Supp Data File 2 Proteomics data analysis.xlsx

Analysis of upregulated phosphopeptides and phosphosites in Eμ-Myc Rel-/- lymphomas versus WT Eμ-Myc lymphomas

Supp Data File 3 Venn diagrams.xlsx

Data files from Venn analysis of Eμ-Myc lymphoma cell proteomics used in this paper

Supp Data File 4 STRING interactions.xlsx

STRING interaction data of the analysis between phosphopeptides and proteins upregulated in c-Rel null versus WT Eμ-Myc lymphomas.

Supp Data File 5 RNASeq_all_genes_list_EuMyc.xlsx

Gene lists from RNA Seq analysis of reimplanted Eμ-Myc lymphoma cells with either vehicle of CHK1i (CCT244747) treatment for 8 hours. Please note, this data file also accompanies two other manuscripts where we use Eμ-Myc lymphoma cells [18, 20].

Supp Data File 6 RNASeq_counts_tximport_EuMyc.xlsx

Data for all genes and samples from RNA Seq analysis of reimplanted Eμ-Myc lymphoma cells. Please note, this data file also accompanies two other manuscripts where we use Eμ-Myc lymphoma cells [18, 20].

Hunter et al., Figure S1

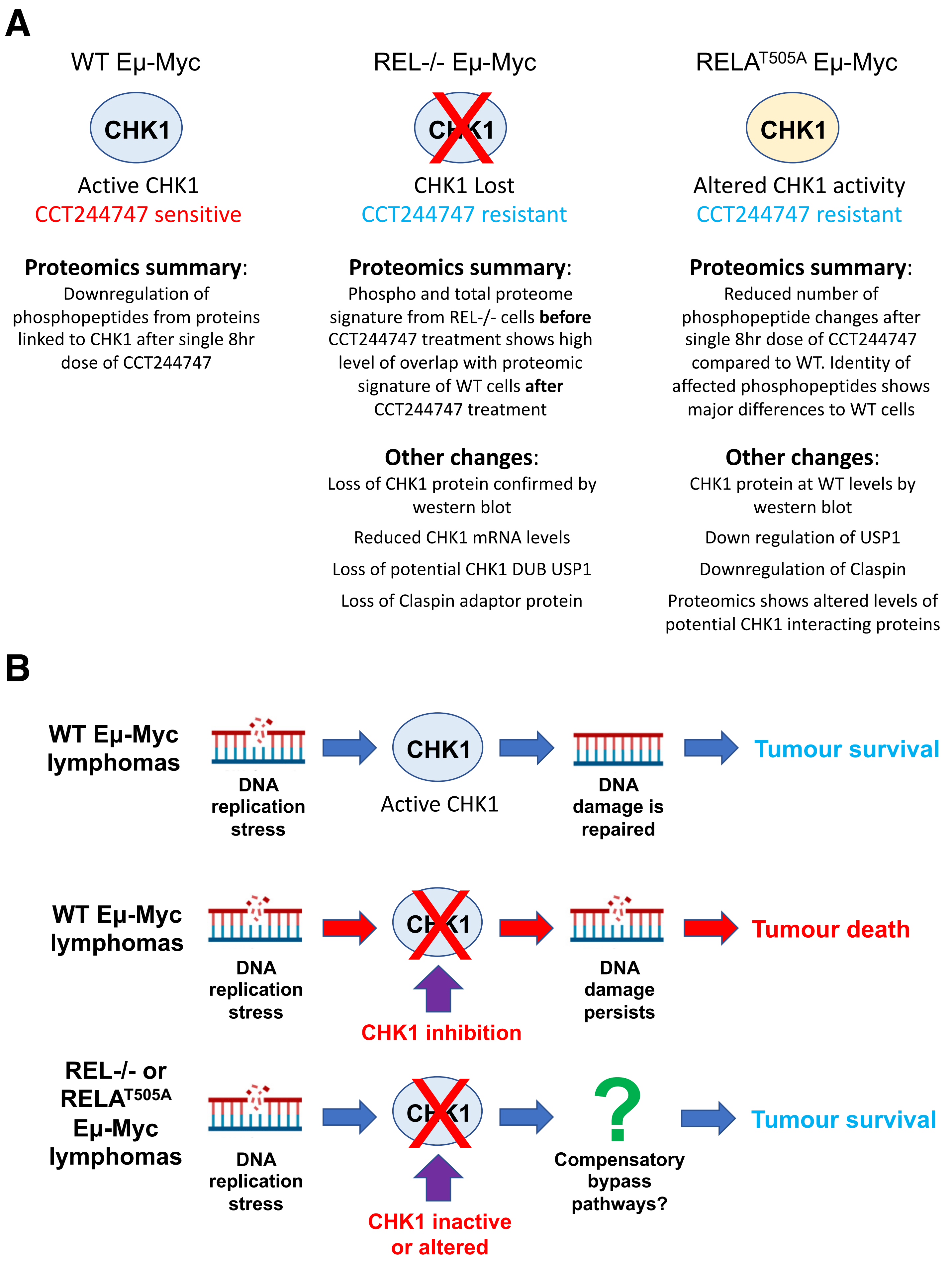

Hunter et al., Figure S2

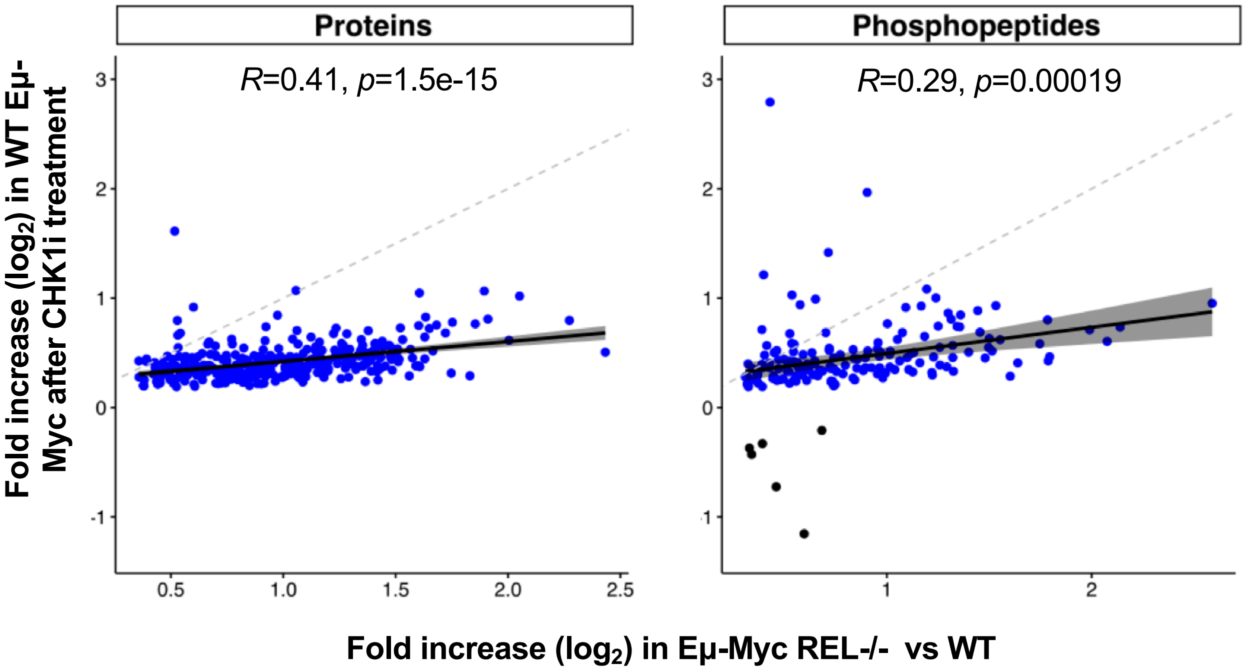

Hunter et al., Figure S3

A

Symbol	Phosphosite (mouse, Eμ-Myc)	Protein Accession	Fold induced in Rel-/- vs WT (log2)	p-value	CST phosphosite(s) (human)	CST name (if different)
Akt1s1	Akt1s1_T247	Q9D1F4	1.19	3.51E-04	T246	PRAS40
Bcl10	Bcl10_S141	Q9Z0H7	0.59	1.79E-02	S218, S231	
Dnmt1	Dnmt1_S125	P13864	0.43	4.37E-02	S143	
Ep300	Ep300_S1037	B2RWS6	0.46	3.20E-02	S1834	p300
Foxo1	Foxo1_S467	Q9R1E0	0.46	2.29E-02	S256, S319, T24	FOXO1a
Foxo1	Foxo1_S284	Q9R1E0	0.34	4.35E-02		
Foxo1	Foxo1_S467	Q9R1E0	0.33	3.31E-02		
Hspb1	Hspb1_S86	P14602	0.87	1.07E-02	S82	HSP27
Palld	Palld_S901	Q9ET54	1.09	1.43E-02	S1118	palladin
Pdcd4	Pdcd4_S94	Q61823	0.93	1.79E-03	S67, S457	
Pea15	Pea15_S116	Q62048	3.14	6.68E-03	S116	
Pea15	Pea15_S116	Q62048	1.31	8.75E-04		
Pea15	Pea15_S116	Q62048	0.74	8.84E-04		
Ranbp3	Ranbp3_S40;S33	Q9CT10	0.50	1.97E-02	S126	
Ranbp3	Ranbp3_S40;S32; S33	Q9CT10	0.44	3.52E-02		
Ranbp3	Ranbp3_S283	Q9CT10	0.40	1.30E-02		
Ranbp3	Ranbp3_[S58/T60]	Q9CT10	0.34	2.30E-02		
Rps3	Rps3_T221	P62908	1.41	1.68E-02	T70	
Usp8	Usp8_S680	Q80U87	0.70	1.77E-02	T945	
Wnk1	Wnk1_S185	P83741	0.80	2.02E-03	T60	
Yap1	Yap1_S149	P46938	0.32	2.74E-02	S127	
Yap1	Yap1_[S46/T48]	P46938	0.31	3.91E-02		
Zyx	Zyx_S336	Q62523	0.44	1.51E-02	S142	zyxin

B

Symbol	Phosphosite(s) (mouse, Eμ-Myc)	Protein Accession	Fold induced in Rel-/- vs WT (log2)	p-value
Aak1	Aak1_S729	Q3UHJ0	0.60	1.12E-02
Bckdk	Bckdk_S31	O55028	0.43	1.18E-02
Bmp2k	Bmp2k_S908	Q91Z96	0.95	6.23E-03
Cdk11b	Cdk11b_S115	P24788	0.68	4.04E-03
Cdk13	Cdk13_[S1146/T1147]	Q69ZA1	0.31	2.56E-02
Map4k1	Map4k1_S370;[S375/Y379]	P70218	0.46	4.20E-02
Map4k4	Map4k4_S701	P97820	0.65	1.73E-03
Mapk14	Mapk14_Y182	P47811	1.09	1.47E-02
Mink1	Mink1_S729	Q9JM52	0.36	3.12E-02
Pi4k2a	Pi4k2a_S47	Q2TBE6	0.39	1.74E-02
Prkab1	Prkab1_S108	Q9R078	0.75	6.22E-03
Prkar1a	Prkar1a_S83	Q9DBC7	1.19	9.02E-03
Prkar2b	Prkar2b_S112	P31324	0.56	4.18E-02
Prpf4b	Prpf4b_Y849	Q61136	0.99	3.29E-02
Snrk	Snrk_S569	Q8VDU5	0.48	2.13E-02
Stk10	Stk10_S437	O55098	0.43	1.37E-02
Stk4	Stk4_S418	Q9JI11	1.53	2.62E-05
Stk4	Stk4_S320	Q9JI11	1.16	1.24E-04
Tnik	Tnik_S737	P83510	0.89	1.52E-03
Wnk1	Wnk1_S185	P83741	0.80	2.02E-03

Hunter et al., Fig. S4

AKT1		ERK1 (MAPK3)		JNK1 (MAPK8)		p38 MAPK (MAPK14)	
**Medium**	**High**	**Medium**	**High**	**Medium**	**High**	**Medium**	**High**
ABI1	ABI1	ABI1	ABI1	BCL10	BCL10	AKAP13	EP300
ACACA	AKT1S1	AKT1S1	BCL10	EP300	EP300	BCL10	FOXO1
AKT1S1	BCL10	BCL10	EP300	FLNA	FLNA	BNIP2	HSPB1
ARHGAP1	CFL1	CANX	FN1	FN1	FOXO1	CDK13	LSP1
ARHGEF6	CRTC2	CFL1	FOXO1	FOXO1	MAPK14	EP300	MEF2C
BCL10	DENND1B	CIC	HSP90AA1	HDAC1	MYC	FLNA	MYC
CANX	DKC1	EP300	HSP90AB1	HSP90AA1	NCOA3	FN1	NCOA3
CD2AP	DOCK2	FLNA	IKZF3	HSPB1	NFATC2	FOXO1	NFATC2
CFL1	EP300	FN1	MAPK14	IRF3	NFKB1	HDAC1	NFKB1
CRTC2	FMNL1	FOXO1	MYC	MAP1B	PXN	HSP90AA1	SLC9A1
DENND1B	FN1	GTF2I	NFATC2	MAPK14	SPAG9	HSP90AB1	SPAG9
DKC1	FOXO1	HDAC1	NFKB1	MYC		HSPB1	
DNMT1	HSP90AA1	HDAC2	PEA15	NCOA3		LCP1	
DOCK2	HSP90AB1	HNRNPK	PRKAR1A	NFATC2		LSP1	
EIF2B5	HSPB1	HSP90AA1	PRKAR2B	NFKB1		MEF2C	
EML4	LAMTOR1	HSP90AB1	PTGES3	PXN		MYC	
EP300	MAPK14	HSPB1	PXN	SPAG9		NCOA3	
EPSTI1	MEF2C	IKZF3	SLC9A1	YAP1		NFATC2	
FLNA	MYC	IRF3	VCL			NFKB1	
FMNL1	NFKB1	MAPK14				PTPRC	
FN1	NPM1	MEF2C				PXN	
FOXO1	PA2G4	MYC				SLC9A1	
GTF2I	PML	NES				SPAG9	
HDAC1	PTGES3	NFATC2					
HDAC2	PXN	NFKB1					
HNRNPA1	SPAG9	PEA15					
HSP90AA1	STUB1	PRKAR1A					
HSP90AB1	USP8	PRKAR2B					
HSPB1	VCL	PTGES3					
IRF3		PTPRC					
LAMTOR1		PXN					
LCP1		SLC9A1					
MAPK14		STUB1					
MARCKS		TRIM28					
MEF2C		VCL					
MYC		YAP1					
MYH9							
NCOA3							
NES							
NFATC2							
NFKB1							
NPM1							
PA2G4							
PACSIN1							
PALLD							
PDCD4							
PEA15							
PML							
PPIG							
PPP1R12A							
PRKAB1							
PTGES3							
PTPRC							
PXN							
SLC9A1							
SLC9A3R1							
SPAG9							
SRSF1							
STK4							
STUB1							
TFEB							
TP53BP1							
USP7							
USP8							
VASP							
VCL							
WDR44							
YAP1							
ZYX							

Hunter et al., Figure S5

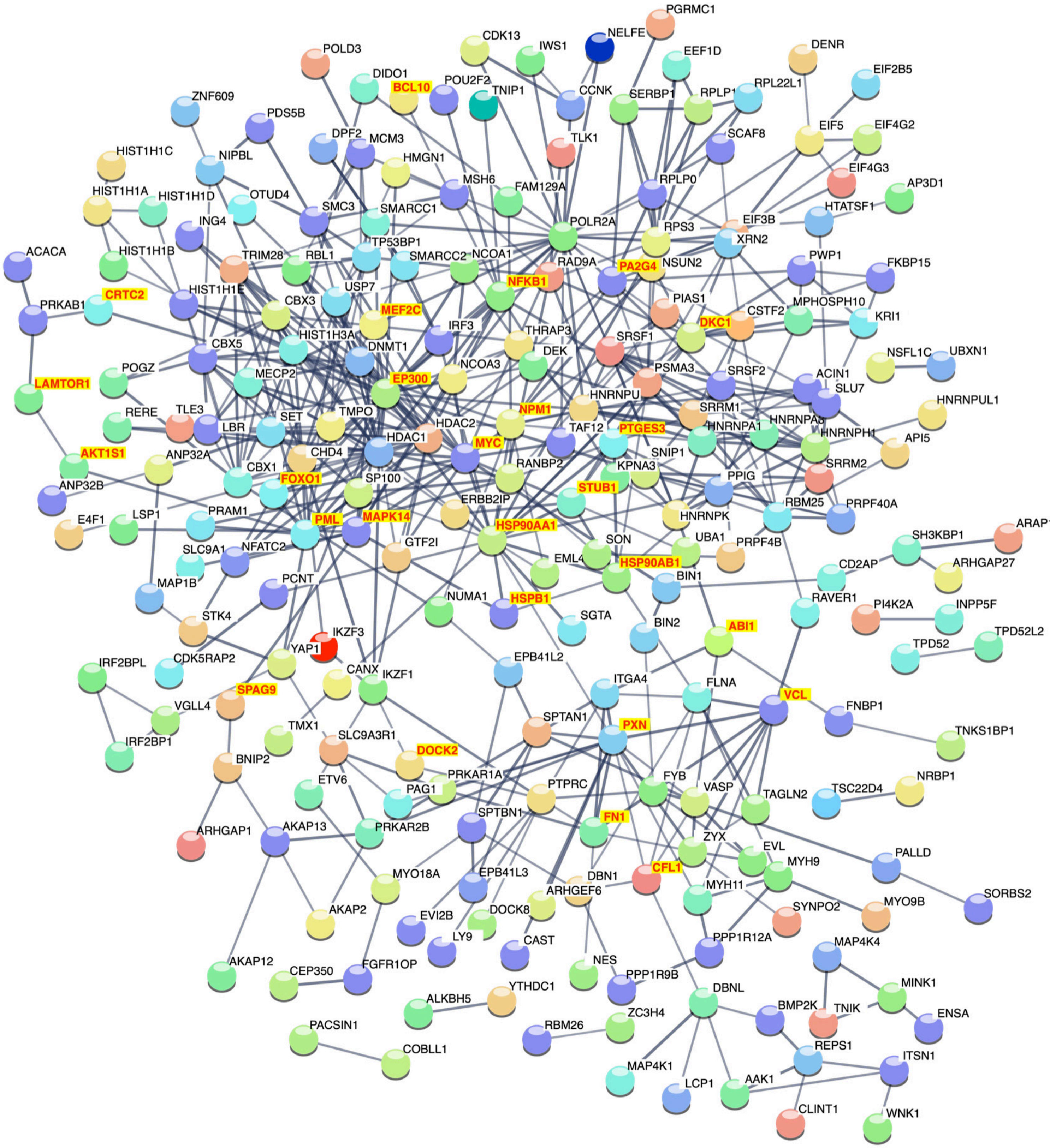

Hunter et al., Figure S6

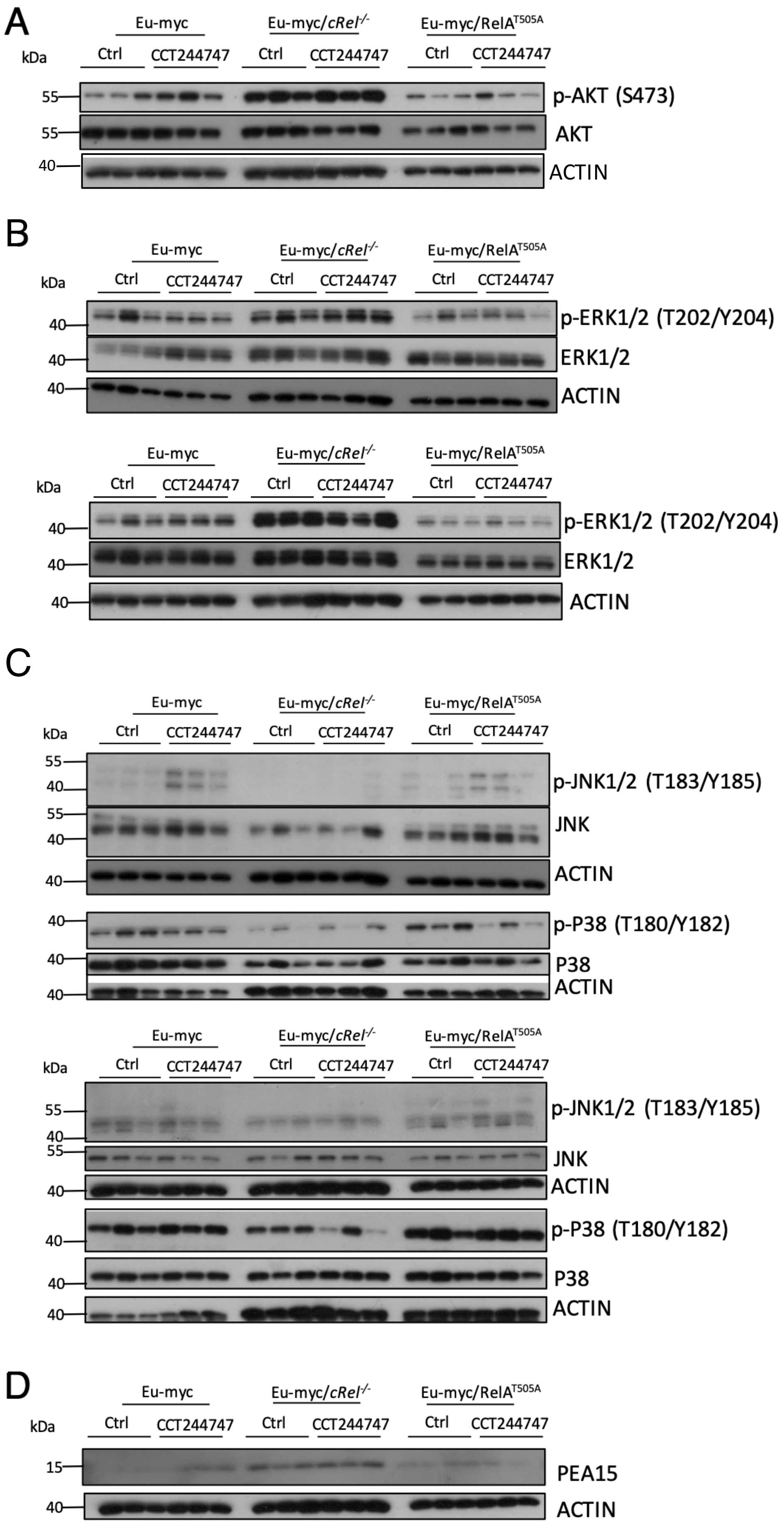

Hunter et al., Figure S7

A

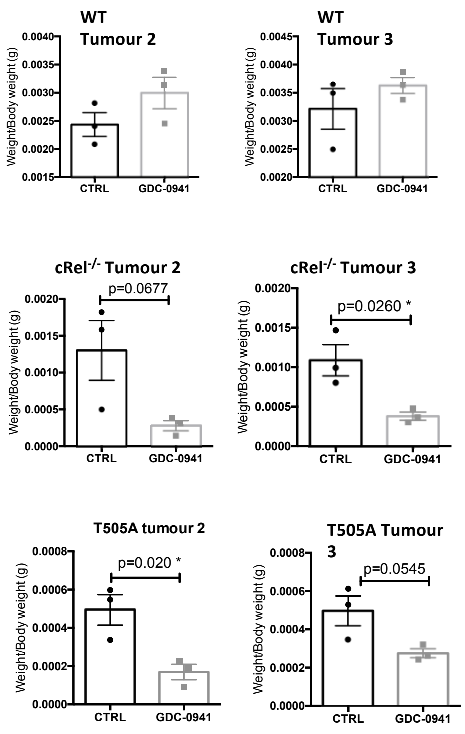

Cervical Lymph node

B

**Tumour**	Lymphoid organ weight/Bodyweight (mg)											
	Inguinal LN		Brachial LN		Mesenteric LN		Cervical LN		Spleen		Thymus	
	Control	GDC-0941	Control	GDC-0941	Control	GDC-0941	Control	GDC-0941	Control	GDC-0941	Control	GDC-0941
**Eu-myc 1**	2.76<br>2.55<br>3.61	3.36<br>2.68<br>2.96	2.36<br>1.24<br>2.97	2.35<br>2.48<br>2.31	11.23<br>5.89<br>8.45	10.09<br>9.68<br>8.97	2.96<br>3.64<br>2.08	5.02<br>3.74<br>2.77	13.10<br>18.73<br>15.19	13.60<br>14.47<br>15.85	5.67<br>8.75<br>4.88	4.78<br>5.07<br>6.91
**Eu-myc 2**	3.74<br>2.78<br>3.23	3.30<br>2.93<br>3.40	2.21<br>1.37<br>3.07	5.10<br>3.39<br>3.84	7.32<br>9.68<br>9.54	7.75<br>7.78<br>12.02	2.81<br>2.08<br>2.40	3.13<br>3.93<br>2.45	18.38<br>20.71<br>17.74	14.34 *<br>14.95<br>15.19	6.81<br>8.90<br>6.24	9.69<br>3.84<br>9.30
**Eu-myc 3**	2.50<br>2.48<br>3.05	2.71<br>3.39<br>3.03	1.78<br>2.13<br>1.98	3.43<br>1.11<br>6.42	5.64<br>8.79<br>6.98	7.70<br>9.72<br>12.26	3.65<br>3.49<br>2.49	3.87<br>3.63<br>3.37	16.74<br>17.00<br>20.50	12.94<br>15.99<br>17.47	6.07<br>6.50<br>6.98	7.48<br>7.32<br>6.38
**Eu-myc/c-Rel-/- 1**	0.44<br>0.31<br>0.35	0.04 *<br>0.27<br>0.06	0.48<br>0.45<br>0.68	0.19 *<br>0.28<br>0.05	1.03<br>1.25<br>1.51	0.98<br>1.30<br>1.07	0.33<br>0.24<br>0.75	0.04 *<br>0.08<br>0.10	5.91<br>4.09<br>10.94	2.15<br>5.80<br>2.49	1.36<br>1.56<br>1.02	1.16<br>1.45<br>2.01
**Eu-myc/c-Rel-/- 2**	2.65<br>1.93<br>3.28	0.57<br>1.97<br>0.75	2.65<br>1.22<br>2.45	0.44<br>1.67<br>0.33	5.78<br>4.09<br>5.73	1.82 *<br>3.86<br>2.57	1.58<br>0.50<br>1.82	0.14<br>0.31<br>0.38	9.40<br>3.09<br>3.76	1.23<br>2.12<br>1.79	3.84<br>3.09<br>3.76	1.23 **<br>2.12<br>1.79
**Eu-myc/c-Rel-/- 3**	0.71<br>1.79<br>2.11	0.52<br>1.10<br>0.89	1.28<br>2.61<br>2.85	0.35 *<br>0.56<br>0.74	3.74<br>3.53<br>4.09	2.38 *<br>3.07<br>2.30	0.80<br>1.47<br>0.99	0.30 **<br>0.37<br>0.48	9.62<br>13.51<br>18.37	6.72<br>8.80<br>8.67	2.61<br>3.77<br>4.29	1.35<br>2.57<br>2.52
**Eu-myc/T505A 1**	0.39<br>0.85<br>0.55	0.18<br>0.45<br>0.28	0.70<br>1.07<br>0.56	0.34<br>0.44<br>0.50	4.99<br>2.09<br>2.34	0.77<br>1.00<br>1.06	0.35<br>0.53<br>0.61	0.26<br>0.32<br>0.24	29.37<br>38.91<br>24.61	27.49<br>35.37<br>18.88	2.61<br>1.35<br>2.52	1.31<br>1.57<br>1.86
**Eu-myc/T505A 2**	0.88<br>1.05<br>0.37	0.38<br>0.34<br>0.34	1.17<br>0.58<br>0.78	0.50<br>0.78<br>0.50	2.25<br>1.99<br>2.80	0.78 **<br>0.92<br>1.18	0.34<br>0.55<br>0.60	0.22 *<br>0.19<br>0.09	27.28<br>25.02<br>30.13	18.22 **<br>18.60<br>18.88	1.86<br>1.30<br>3.58	1.68<br>1.39<br>1.43
**Eu-myc/T505A 3**	0.77<br>0.68<br>0.77	0.28 *<br>0.41<br>0.52	0.81<br>0.57<br>0.69	0.30 *<br>0.38<br>0.45	1.44<br>1.39<br>1.12	1.06<br>1.57<br>0.78	0.65<br>0.49<br>0.50	0.10 **<br>0.08<br>0.10	31.37<br>26.90<br>35.77	25.57 *<br>19.12<br>19.53	1.69<br>2.25<br>1.56	1.30<br>1.88<br>1.86

Hunter et al., Figure S8

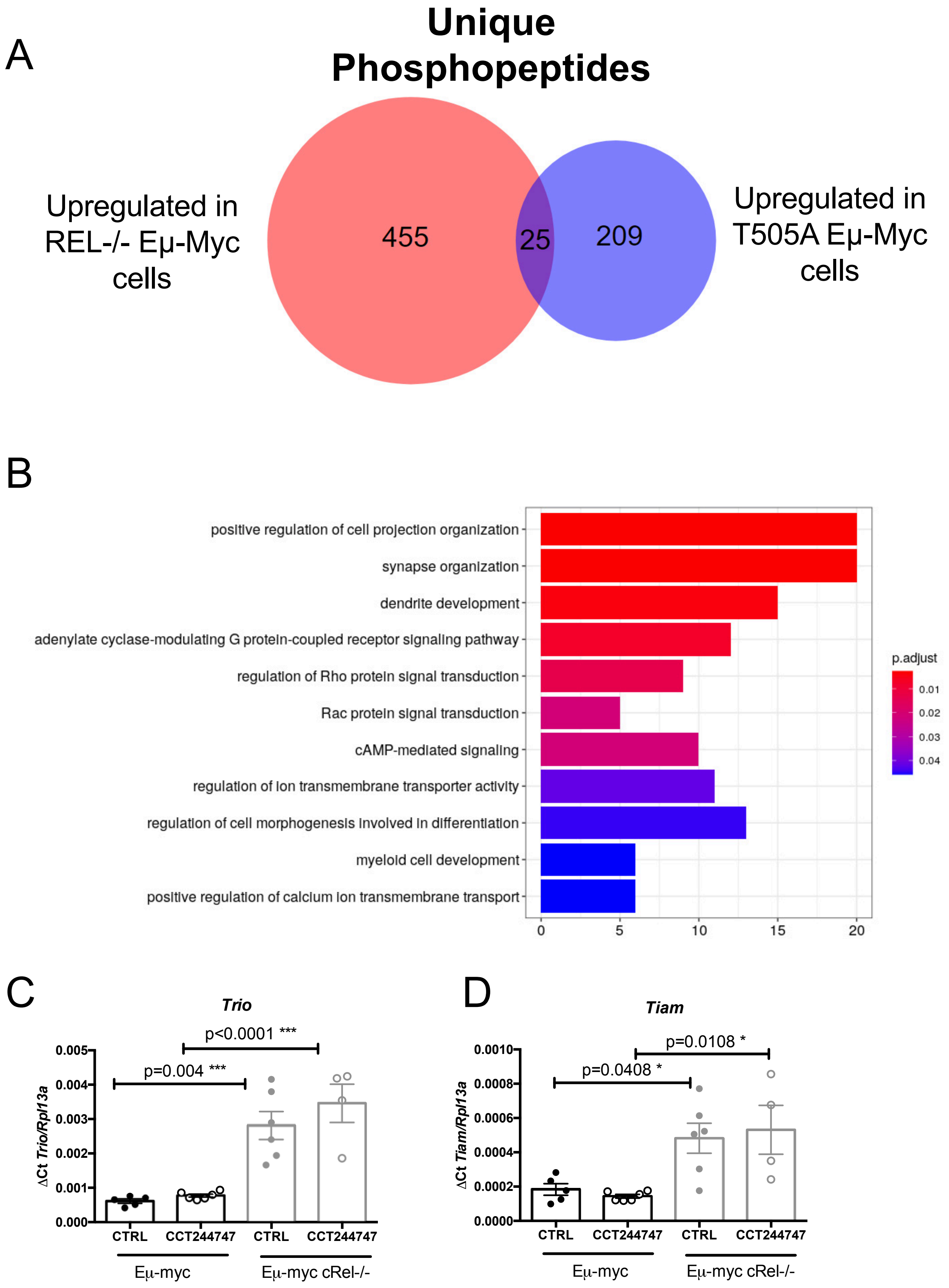

Hunter et al., Figure S9

A

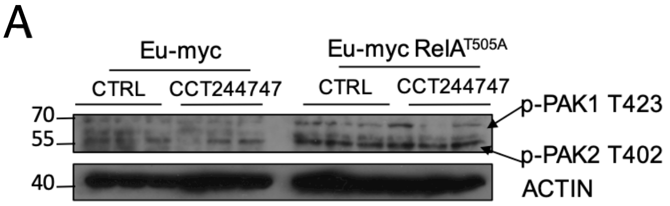

B

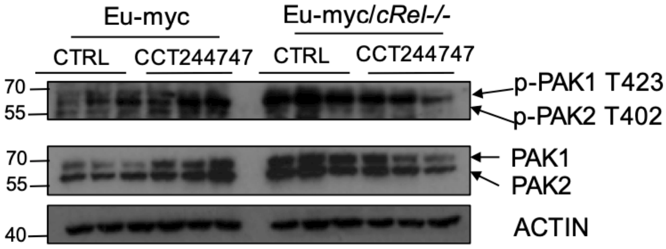

C

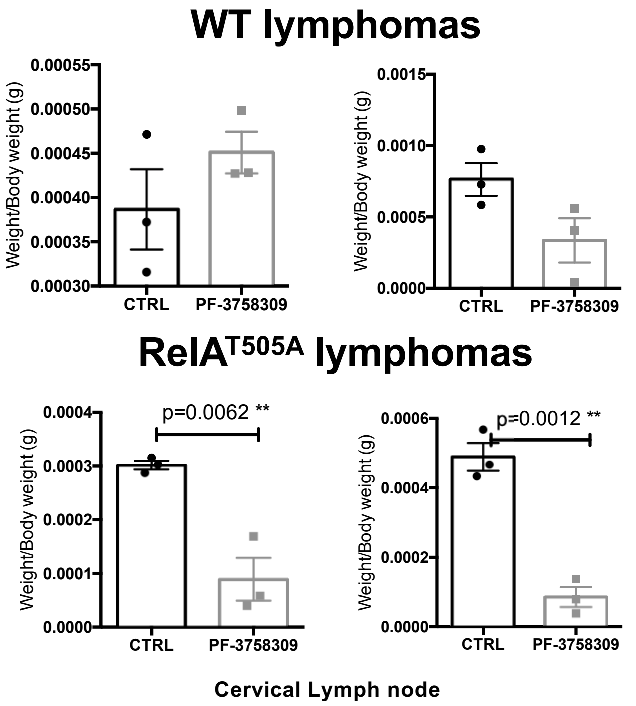

D

**Tumour**	**Lymphoid organ weight/Bodyweight (mg)**											
	**Inguinal LN**		**Brachial LN**		**Mesenteric LN**		**Cervical LN**		**Spleen**		**Thymus**	
	Control	PF3758309	Control	PF3758309	Control	PF3758309	Control	PF3758309	Control	PF3758309	Control	PF3758309
**Eu-myc 1**	0.65<br>0.54<br>0.39	0.62<br>0.65<br>0.42	1.05<br>0.86<br>0.73	0.46<br>0.78<br>0.68	2.24<br>1.82<br>2.21	0.94<br>2.15<br>1.87	0.73<br>0.58<br>0.98	0.56<br>0.04<br>0.41	9.84<br>8.95<br>9.12	6.59<br>12.99<br>7.22	2.15<br>1.94<br>2.19	1.18<br>1.97<br>2.51
**Eu-myc 2**	1.00<br>0.70<br>1.46	0.58<br>0.57<br>0.78	1.20<br>1.11<br>0.75	0.55<br>0.77<br>0.63	2.50<br>2.78<br>2.33	2.07<br>2.83<br>1.68	0.67<br>0.63<br>0.41	0.37<br>0.36<br>0.62	7.32<br>8.41<br>10.62	7.72<br>8.47<br>7.52	1.28<br>1.79<br>4.51	1.68<br>1.26<br>1.84
**Eu-myc 3**	0.92<br>0.88<br>1.48	0.92<br>1.42<br>0.86	1.18<br>1.25<br>0.81	1.10<br>0.90<br>1.16	2.38<br>1.68<br>3.48	1.86<br>2.16<br>2.08	0.32<br>0.37<br>0.47	0.43<br>0.43<br>0.50	7.74<br>8.77<br>13.75	6.89<br>7.08<br>7.55	1.25<br>1.79<br>1.58	1.50<br>2.00<br>1.28
**Eu-myc/T505A 1**	0.93<br>0.48<br>1.14	0.29 *<br>0.08<br>0.15	0.45<br>0.85<br>0.79	0.23 *<br>0.13<br>0.17	1.45<br>1.43<br>1.46	0.83 **<br>0.79<br>1.05	0.47<br>0.43<br>0.57	0.14<br>0.08<br>0.04	5.20<br>4.96<br>6.01	5.99 **<br>5.52<br>5.75	2.71<br>2.19<br>2.03	0.57 **<br>0.62<br>0.63
**Eu-myc/T505A 2**	0.75<br>0.38<br>0.88	0.29 *<br>0.14<br>0.28	0.42<br>0.87<br>0.74	0.18<br>0.17<br>0.27	1.57<br>1.96<br>1.20	0.68<br>0.88<br>1.14	0.43<br>0.30<br>0.36	0.12<br>0.05<br>0.11	5.27<br>4.19<br>4.91	5.76 **<br>4.63<br>5.27	2.19<br>1.85<br>1.69	0.65 **<br>0.74<br>0.80
**Eu-myc/T505A 3**	0.91<br>0.73<br>0.77	0.11 ***<br>0.06<br>0.18	0.60<br>0.62<br>0.56	0.16 ***<br>0.25<br>0.12	1.58<br>1.37<br>1.63	1.12 *<br>1.00<br>0.71	0.29<br>0.32<br>0.30	0.04<br>0.17<br>0.06	3.69<br>4.09<br>5.46	4.32 *<br>8.43<br>7.08	1.49<br>1.47<br>1.88	1.17 **<br>0.76<br>0.81
